# Medium Adaptation and Function Extension of WGM Optical Microcavity Acoustic Sensing: A Review of Air-Coupled and Liquid-Phase Detection, Photoacoustic Imaging, and Multifunctional Sensing

**DOI:** 10.3390/s26216916

**Published:** 2026-10-31

**Authors:** Rong Wang, Wenqing Liu, Qian Li, Pengcheng Shi, Jianning Han

**Affiliations:** School of Physics and Electronic Engineering, Shanxi Normal University, Taiyuan 030031, China; 20250014@sxnu.edu.cn (R.W.); lwq2232194531@163.com (W.L.); 20250085@sxnu.edu.cn (Q.L.); shipengchengzb@163.com (P.S.)

**Keywords:** whispering gallery mode, optical microcavity, acoustic sensing, photoacoustic imaging, multifunctional sensing

## Abstract

Whispering-gallery-mode (WGM) optical microcavity acoustic sensors have attracted extensive attention owing to their high sensitivity, broad bandwidth, miniaturization and anti-electromagnetic interference capability. This review systematically surveys medium-adapted acoustic sensing and function extension applications of WGM microcavities. Starting from perturbation theory, we distinguish between optical quality factor *Q*_0_ and mechanical quality factor *Q_m_*, and elucidate the intrinsic sensitivity–bandwidth trade-off dominated by mechanical dynamics. We summarize advances in air-coupled and liquid-phase acoustic detection, photoacoustic imaging, and multiparameter multifunctional sensing. Practical bottlenecks including calibration deficiency, thermal drift-induced crosstalk, packaging trade-offs, long-term stability and array scalability are analyzed. Joint hardware–process–algorithm optimization strategies are discussed, providing references for advancing WGM microcavity acoustic sensors toward real-world engineering deployment.

## 1. Introduction

Acoustic sensors have been widely applied in fields such as biomedical imaging [1,2,3,4], underwater detection [5], industrial non-destructive testing [6,7], and health monitoring [8]. Whispering-gallery-mode (WGM) optical microcavity acoustic sensors employ an optical microcavity as the core sensing element and demodulate acoustic signals by detecting resonant mode variations induced by acoustic perturbations. Owing to their outstanding features, including ultra-high detection sensitivity, broadband acoustic response, miniaturization, and immunity to electromagnetic interference, they have attracted increasing attention in high-sensitivity acoustic sensing in recent years.

As the requirements for comprehensive sensing performance in acoustic detection scenarios continue to rise, conventional piezoelectric [9,10,11] and microelectromechanical system (MEMS) [12,13,14,15] acoustic sensors, although technologically mature, suffer from degraded sensitivity upon miniaturization, along with inherent limitations such as bandwidth constraints and susceptibility to electromagnetic interference, making it difficult for them to fully satisfy high-performance detection demands. WGM optical microcavity acoustic sensing [16,17,18,19,20,21,22,23,24,25,26] exhibits clear advantages in these respects. Nevertheless, its engineering deployment still faces numerous practical obstacles, including the absence of acoustic pressure calibration standards, acoustic impedance mismatch, multi-physical-field coupling crosstalk, packaging constraints, long-term operational stability, and array-scale readout, which jointly restrict the transition of this technology from laboratory proof-of-concept to practical applications.

At present, numerous reviews have discussed WGM optical microcavities [27,28,29,30,31,32,33,34,35,36,37,38,39,40,41,42,43,44,45,46,47,48,49,50], covering a wide range of applications, including biosensing, temperature sensing, optofluidic detection, displacement sensing, mass sensing, magnetic sensing, and acoustic sensing. Among them, Ref. [48] compared the ultrasound-sensing performance of three types of optical microcavities, namely Fabry–Pérot cavities, π-phase-shifted Bragg gratings, and WGM microcavities, while Ref. [49] focused on the physical mechanisms underlying acousto-optical interactions inside three-dimensional WGM resonators.

Nevertheless, reviews dedicated solely to WGM microcavity acoustic sensing [43,44,45,46,47,48,49,50] remain scarce. The existing literature provides few systematic syntheses of research progress across different acoustic propagation media, nor does it offer consolidated discussions on noise–bandwidth trade-offs, practical engineering bottlenecks, and advances in multifunctional sensing. The disparities in content coverage among these representative reviews are summarized in Table 1.

A systematic literature search was conducted for this review across Web of Science, Scopus, and Google Scholar for publications published between 2010 and 2026. Device-related keywords (“whispering gallery mode”, “optical microcavity”, “microresonator”) and application-oriented keywords (“acoustic sensing”, “ultrasound detection”, “photoacoustic”, “hydrophone”) were combined using Boolean operators for retrieval. In terms of literature screening, peer-reviewed journal articles were given priority. Conference abstracts, short preliminary reports and non-peer-reviewed resources were excluded. Representative high-impact studies published in the past five years were preferentially included to reflect the latest developments in this field.

Building on the above analysis, the structure of this review is organized as follows. First, it elaborates on acoustic sensing mechanisms and readout schemes of WGM microcavities. It then analyzes the noise–bandwidth trade-offs via the noise-equivalent pressure (NEP). Subsequent sections summarize medium-adapted sensing advances under air-coupled and liquid-phase conditions, as well as developments in photoacoustic imaging and multifunctional sensing applications. Finally, core technical bottlenecks are analyzed and future research directions are discussed, aiming to provide a reference for subsequent research in this field.

## 2. WGM Optical Microcavity

**Figure 1:**
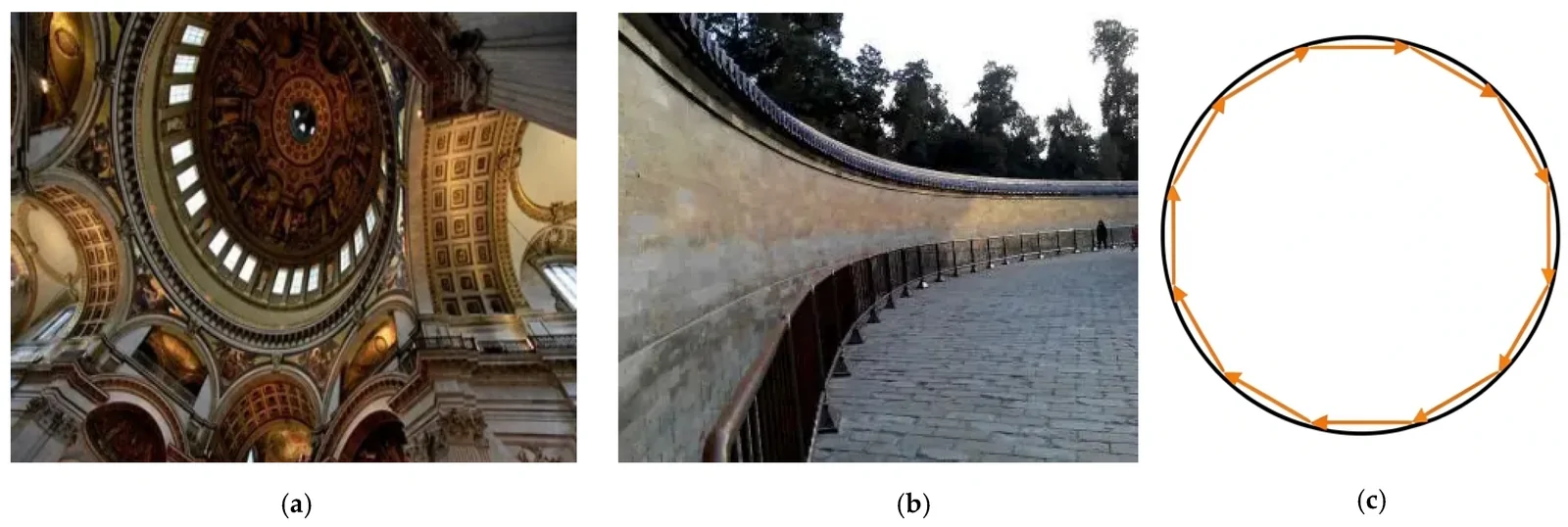
A schematic illustration of the whispering gallery phenomenon and total internal reflection. (**a**) The whispering gallery in St Paul’s Cathedral, London. (**b**) The echo wall at the Temple of Heaven, Beijing. (**c**) Total internal reflection in a WGM optical microcavity.

The concept of whispering gallery mode originated in the field of acoustics. In 1910, Lord Rayleigh systematically studied the phenomenon of long-distance propagation of sound waves through continuous low-loss reflections along the curved walls of the whispering gallery in St. Paul’s Cathedral in London [51] (Figure 1a). Similarly, the echo wall at the Temple of Heaven in Beijing exhibits a comparable acoustic effect (Figure 1b). This unique acoustic phenomenon was named the “whispering gallery mode”. Later, researchers extended this physical concept to the field of optics. When light propagates from an optically denser medium to an optically rarer medium, and the angle of incidence exceeds the critical angle, total internal reflection occurs (Figure 1c). WGM optical microcavities confine light within an extremely small mode volume at the mesoscopic scale and exhibit ultra-high quality factors (Q factors), thereby greatly enhancing light–matter interactions.

**Figure 2:**
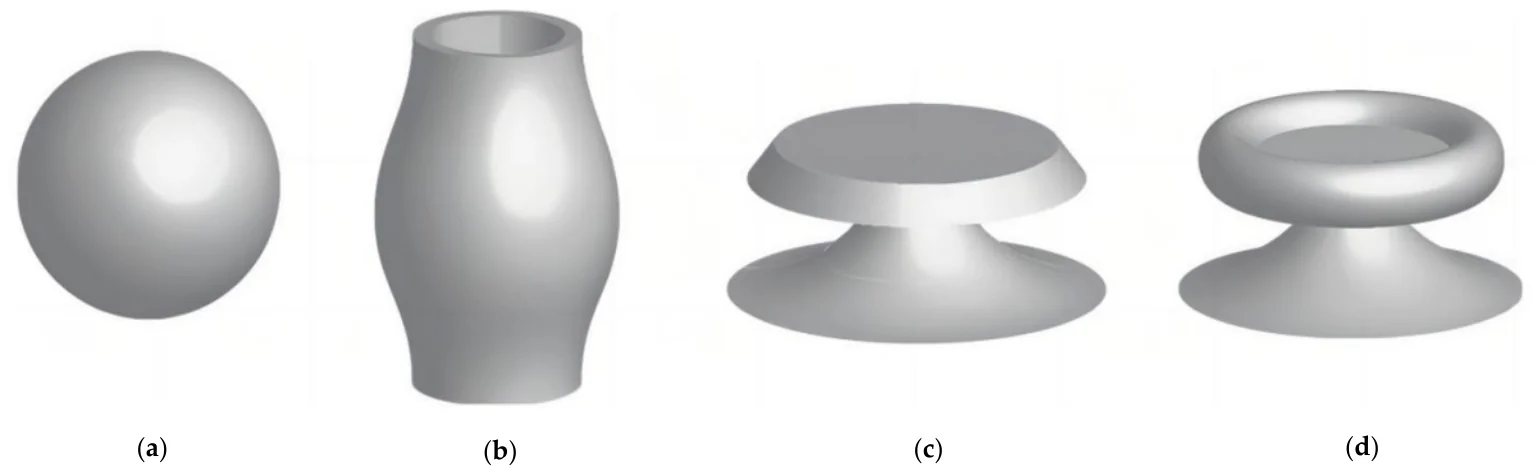
Schematic illustrations of WGM optical microcavities with different geometric configurations [32]. (**a**) Microsphere cavity; (**b**) microbubble cavity; (**c**) microdisk cavity; (**d**) microtoroid cavity.

To meet the demands of a wide array of applications, existing WGM optical microcavities exhibit diverse geometric configurations [32] (Figure 2a–d). Common geometries include microsphere cavities [52,53,54,55], microdisk cavities [17,56,57], microring cavities [26,58,59,60], microtoroid cavities [61,62,63], microbubble cavities [18,64,65,66,67], microbottle cavities [68,69,70,71], microknot cavities [72,73], micropillar cavities [74,75], microtube cavities [76,77], and polygonal microcavities [78], among others. Variations in geometry strongly influence several key intrinsic properties of the cavity, including the Q factor, mode volume, mechanical responsivity, and coupling interface condition. These properties, in turn, provide an important physical basis for the medium-adapted acoustic sensing and multifunctional sensing discussed in this review.

Moreover, the performance of WGM optical microcavities depends not only on geometric configuration but also, to a large extent, on the material properties. To achieve effective total internal reflection and further reduce optical losses, the materials used to fabricate these devices are usually required to possess a high refractive index, a broad transparency window, an ultra-smooth surface, and a low thermo-optic coefficient, as well as intrinsic mechanical and thermo-optical properties critical for acoustic response characteristics. Common material systems for WGM microcavities cover amorphous inorganic glasses (e.g., SiO_2_ [54,61,79,80], chalcogenide glasses [81,82,83,84]), inorganic dielectrics (e.g., Si_3_N_4_ [26,59,85,86]), crystalline materials including semiconductor crystals (Si [87,88,89,90], GaAs [91,92], InP [93,94]), dielectric crystals (CaF_2_ [95,96,97], MgF_2_ [98,99], LiNbO_3_ [100,101]), and polymers [69,102,103,104,105] (e.g., PMMA [106,107], PS [108,109,110], SU-8 [111], PDMS [112,113]). Each material family exhibits its own compromises among optical, mechanical, and thermal properties, which largely govern its suitability for diverse acoustic sensing scenarios. The advantages and limitations of these typical materials are summarized in Table 2.

There exist inherent performance trade-offs among different material families. Some materials can realize ultra-high-Q resonances but face challenges in on-chip array implementation. Others exhibit excellent integration capability, yet are restricted by intrinsic optical loss. Material-dependent thermo-optic and elasto-optic parameters constitute important limiting factors. Such material-related intrinsic features interact with external propagation media and multi-physical fields.

Taken together, WGM optical microcavities exhibit marked differences in optical loss, mode volume, mechanical stiffness, photoelastic properties, environmental robustness, and integration capability across different geometric configurations and materials. The wall thickness, geometric dimensions, and intrinsic material parameters jointly determine the acoustic response of the cavity, which provides the basis for the geometric design and performance optimization of WGM acoustic sensors and sets the stage for the medium-adapted acoustic sensing and multifunctional sensing discussed in subsequent sections.

## 3. Mechanisms and Performance Fundamentals of WGM Optical Microcavity Acoustic Sensing

**Figure 3:**
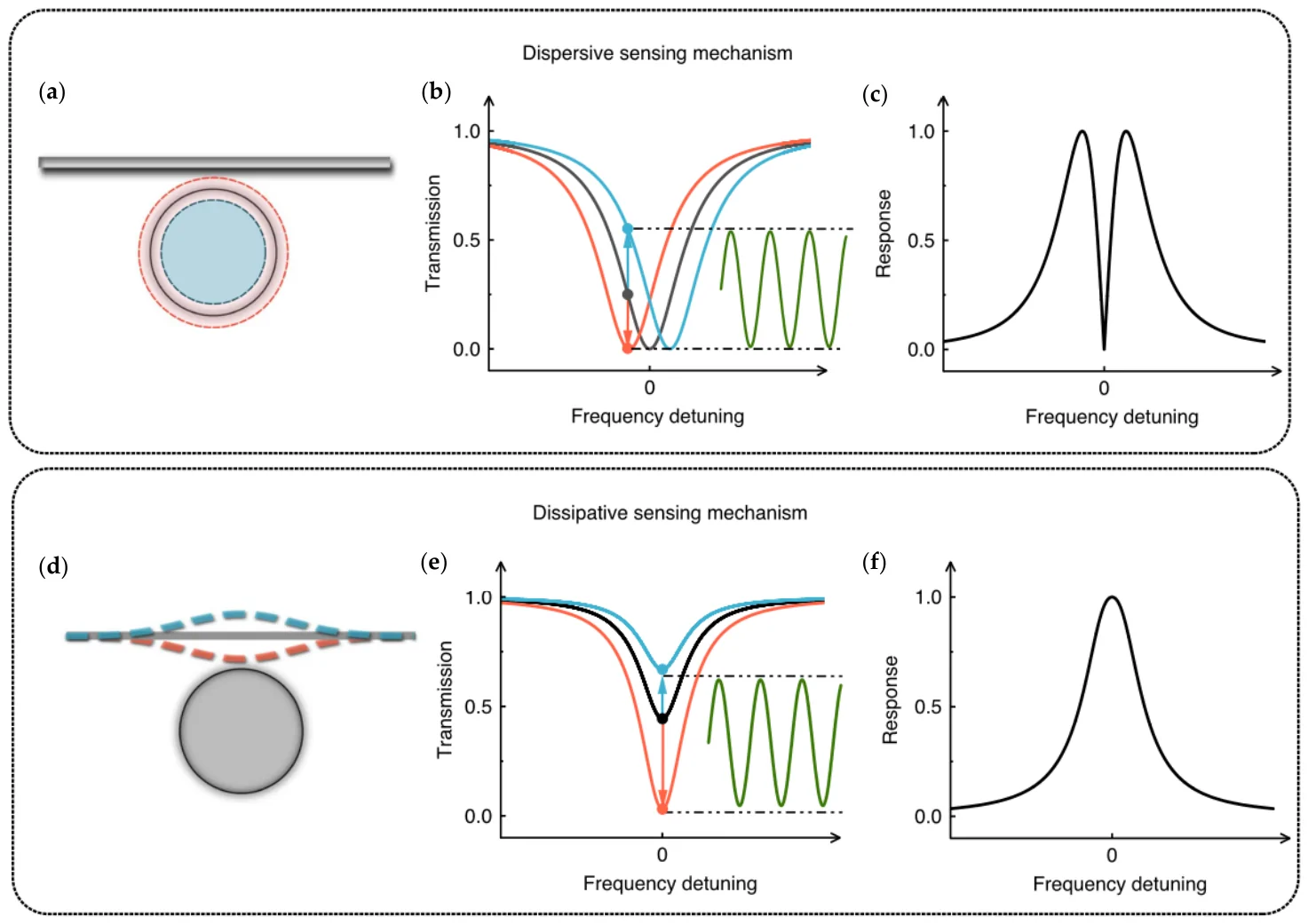
Acoustic sensing mechanisms [48]. (**a**–**c**) The dispersive coupling sensing mechanism; (**d**–**f**) the dissipative coupling sensing mechanism. (**a**,**d**) Schematic illustrations of the dispersive and dissipative coupling sensing mechanisms in an optical microcavity, respectively; (**b**,**e**) transmission spectral variations in the optical microcavity under dispersive and dissipative coupling interactions, respectively. Black line: transmission spectrum without acoustic perturbation; blue and red lines: spectra under acoustic perturbation; green oscillating curve: modulated readout signal. (**c**,**f**) Signal response curves of the dispersive and dissipative coupling sensing mechanisms, respectively.

The operating principle of WGM optical microcavity acoustic sensing is that acoustic pressure deforms the microcavity structure, alters the material refractive index, or modifies the waveguide–microcavity coupling condition. These perturbations are read out optically through variations in the resonance frequency, transmitted intensity, or resonance linewidth. Depending on how the acoustic signal modulates the optical response, the sensing mechanisms can be broadly classified into dispersive coupling [114,115,116,117,118,119] and dissipative coupling [120,121,122,123]. The former manifests primarily as a shift in the optical resonance frequency. The latter appears mainly as a change in optical loss or the external coupling rate. In practice, both mechanisms may coexist in the same device [124]. Figure 3 schematically illustrates these two sensing mechanisms [48]).

### 3.1. Fundamentals of Acoustic Pressure-Induced Optical Perturbation

The resonance condition of a WGM optical microcavity can be approximately expressed as [125]

(1) $$m\lambda_{0}=2\pi n_{eff}R,$$

where *m* is the azimuthal mode number, $\lambda_{0}$ is the resonant wavelength in vacuum, $n_{eff}$ is the effective refractive index of the mode, and R is the cavity radius. When an external acoustic pressure is applied to the microcavity, it simultaneously induces elastic geometric deformation of the cavity and refractive index modulation arising from the photoelastic effect. Under the small-perturbation approximation, applying a first-order perturbation to Equation (1) yields the relative shift in the resonant angular frequency [126]:

(2) $$\frac{\Delta \omega_{0}}{\omega_{0}}=-\frac{\Delta R}{R}-\frac{\Delta n_{eff}}{n_{eff}}$$

where $\omega_{0}$ is the unperturbed optical resonant angular frequency, $\Delta \omega_{0}$ is the frequency shift induced by acoustic pressure, ΔR is the change in the cavity radius, and $\Delta n_{eff}$ is the change in the effective refractive index. This relation indicates that acoustic pressure modulation of the resonance characteristics consists of two contributions: geometric deformation and refractive index variation. The refractive index change induced by acoustic pressure is dominated by the photoelastic effect. Based on the constitutive relation of the photoelastic effect [127], and adopting the scalar approximation of the effective photoelastic coefficient, the dependence of the material refractive index on mechanical strain can be approximately written as

(3) $$\Delta n=-\frac{1}{2}n^{3}p_{eff}S,$$

where Δn is the change in the material refractive index, n is the refractive index of the material, $p_{eff}$ is the effective photoelastic coefficient, and S is the mechanical strain. It should be noted that Δn in Equation (3) denotes the change in the material refractive index, which is related to but not strictly identical to the change in the effective refractive index $\Delta n_{eff}$ in Equation (2); under the first-order approximation for WGM, the relative change $\Delta n_{eff}/n_{eff}$ is approximately equal to Δn/n. Equations (2) and (3) show that acoustic pressure determines the resonance frequency shift through the combined action of mechanical deformation and the photoelastic effect.

To establish a quantitative relationship between the acoustic pressure and the mechanical response, the $e^{-i\Omega t}$ time factor convention is adopted. Under the single-mechanical-mode linear-response approximation, the mechanical response function can then be expressed as [128]

(4) $$\chi_{m}\left(\Omega \right)=\frac{1}{m_{eff}\left({\Omega_{m}}^{2}-\Omega^{2}-i\Gamma_{m}\Omega \right)},$$

where Ω is the angular frequency of the acoustic excitation, $m_{eff}$ is the effective mechanical mass, $\Omega_{m}$ is the mechanical resonant angular frequency, and $\Gamma_{m}$ is the mechanical damping rate. The acoustic pressure can be equivalently treated as a generalized force $F_{ac}\left(\Omega \right)=A_{eff}p\left(\Omega \right)$, where $A_{eff}$ is the effective pressure area that characterizes the conversion from acoustic pressure to the mechanical generalized force, with units of m^2^, and $p\left(\Omega \right)$ is the frequency-domain component of the acoustic pressure. By further defining the coupling coefficient between the optical resonance frequency and the mechanical displacement $G=\partial \omega_{0}/\partial x$, which incorporates both the geometric deformation and photoelastic contributions of the mechanical mode, the conversion from acoustic pressure to the optical resonance frequency shift can be written as

(5) $$\Delta \omega_{0}\left(\Omega \right)=G\chi_{m}\left(\Omega \right)A_{eff}p\left(\Omega \right).$$

Equation (5) shows that the conversion from acoustic pressure to the optical frequency shift depends not only on the optomechanical coupling coefficient but also on the mechanical response characteristics. For mechanical resonance-enhanced structures, a higher mechanical quality factor $Q_{m}=\Omega_{m}/\Gamma_{m}$ generally enhances the response near resonance. However, it also narrows the mechanical response bandwidth. Besides acoustic pressure, external perturbations such as temperature and refractive index also induce resonance drift. Its general form can be written as $\Delta \omega_{0}=\sum_{i}\left(\partial \omega_{0}/\partial \xi_{i}\right)\Delta \xi_{i}$, where $\xi_{i}$ is the *i*-th external perturbation parameter and $\Delta \xi_{i}$ is its variation.

### 3.2. Readout Mechanisms for Dispersive and Dissipative Coupling

In dispersive coupling, acoustic pressure shifts the resonance frequency by changing the cavity dimensions, refractive index, or mode distribution, and this shift is subsequently converted into a change in the transmitted optical intensity. Let $\omega_{L}$ be the laser angular frequency and $\Delta =\omega_{L}-\omega_{0}$ be the detuning. Under the small-signal approximation, and assuming a fixed laser frequency and a quasi-static optical response, the transmitted intensity variation can be written as

(6) $$\Delta T_{\mathrm{disp}}=-\frac{\partial T}{\partial \Delta }\Delta \omega_{0},$$

where T is the normalized transmittance and ∂T/∂Δ is the transmission slope at the operating point. In practice, the laser is biased at an operating point with a steep resonance slope. This maximizes the efficiency of converting frequency modulation into intensity variation. The optical quality factor is defined as $Q_{0}=\omega_{0}/\kappa$, where $\kappa =\kappa_{i}+\kappa_{e}$ is the total optical energy decay rate, $\kappa_{i}$ is the intrinsic optical energy decay rate, and $\kappa_{e}$ is the external coupling loss rate. A higher optical quality factor $Q_{0}$ corresponds to a narrower resonance linewidth, which improves the optical frequency resolution. However, the minimum detectable acoustic pressure ultimately depends on multiple factors, including the mechanical response amplitude, laser frequency noise, and detector noise. Therefore, a higher optical quality factor $Q_{0}$ does not necessarily lead to higher sensitivity. Dispersive coupling is well suited to structures in which the resonance frequency shift can be stably read out, and sufficient mechanical response is available within the target acoustic band. Its main limitations include environmental drift, laser frequency noise, and the readout-chain bandwidth.

Unlike dispersive coupling, dissipative coupling does not rely on direct measurement of the resonance frequency shift. Instead, it detects acoustic signals through changes in the optical loss or the waveguide–microcavity coupling state induced by acoustic pressure. When acoustic pressure modifies the coupling gap g,the change in the external coupling loss rate can be approximately expressed as $\Delta \kappa_{e}\approx \left(\partial \kappa_{e}/\partial g\right)\Delta g$. Considering the intrinsic loss rate variation $\Delta \kappa_{i}$, and under the same small-signal and quasi-static optical response approximations as in Equation (6), the transmission response is given by the following:

(7) $$\Delta T_{diss}=\frac{\partial T}{\partial \kappa_{i}}\Delta \kappa_{i}+\frac{\partial T}{\partial \kappa_{e}}\Delta \kappa_{e}$$

The dissipative response is closely related to the coupling conditions, the intrinsic loss, and the operating point. Previous studies have shown that, under specific device and coupling conditions, the dissipative acousto-optic interaction can produce a strong response with a weak apparent dependence on the optical quality factor $Q_{0}$ [24]. However, this finding cannot be generalized to conclude that dissipative coupling is completely independent of the optical quality factor $Q_{0}$. Dissipative coupling offers a potential route for broadband acoustic detection. However, its detection limit remains constrained by the coupling conditions, resonance characteristics, operating point, and laser and detector noise.

### 3.3. Noise, Bandwidth and Performance Limit Constraints

In practical microcavities, dispersive and dissipative modulations can coexist. When the laser frequency is fixed near the static operating point, and under the first-order small-signal and quasi-static optical response approximations, the total transmittance variation can be uniformly expressed as follows, with all partial derivatives evaluated at the static operating point:

(8) $$\Delta T\approx -\frac{\partial T}{\partial \Delta }\Delta \omega_{0}+\frac{\partial T}{\partial \kappa_{i}}\Delta \kappa_{i}+\frac{\partial T}{\partial \kappa_{e}}\Delta \kappa_{e}$$

Following the definition of the noise-equivalent pressure spectral density, the output voltage noise is referred to the input acoustic pressure port. The noise-equivalent pressure spectral density, hereafter abbreviated as NEP, is then defined as follows:

(9) $$\mathrm{NEP}\left(f\right)=\frac{\sqrt{S_{V}\left(f\right)}}{\left|H_{VP}\left(f\right)\right|}$$

where $S_{V}\left(f\right)$ is the single-sided power spectral density of the output voltage noise, with units of V^2^/Hz, $H_{VP}\left(f\right)$ is the frequency-domain transfer function from the acoustic pressure to the output voltage, with units of V/Pa, and f is the frequency; the unit of $NEP\left(f\right)$ is Pa/Hz^1/2^. This definition unifies the expressions of sensitivity and noise used in different publications, facilitating direct comparison across different devices. For a linear mechanical mode satisfying the classical thermal-equilibrium condition, mechanical thermal noise is an important intrinsic noise source that limits the detection performance. Its single-sided power spectral density of the mechanical Brownian force can be expressed as follows [128]:

(10) $$S_{FF,th}=4k_{B}T_{env}m_{eff}\Gamma_{m}$$

where $k_{B}$ is the Boltzmann constant, and $T_{env}$ is the ambient absolute temperature. The corresponding thermal noise limit can be expressed as follows:

(11) $${\mathrm{NEP}}_{th}=\frac{\sqrt{4k_{B}T_{env}m_{eff}\Gamma_{m}}}{\left|A_{eff}\right|}$$

It should be noted that the thermal noise-limited NEP in Equation (11) is frequency-independent, because the mechanical susceptibility $\chi_{m}\left(\Omega \right)$ cancels between the noise and signal paths. However, when laser frequency noise, detector noise, or other non-thermal noise sources dominate, the total NEP recovers its frequency dependence through Equation (9). Substituting $\chi_{m}\left(\Omega \right)$ from Equation (4) into the transfer function, and assuming a frequency-independent detector noise floor, yields an explicit angular-frequency form, $NEP\left(\Omega \right)=C\sqrt{{\left({\Omega_{m}}^{2}-\Omega^{2}\right)}^{2}+{\left(\Gamma_{m}Q_{m}\right)}^{2}}$. Substituting Ω=2πf and $Q_{m}=\Omega_{m}/\Gamma_{m}$, this becomes

(12) $$\mathrm{NEP}\left(f\right)=C^{\prime }\sqrt{{\left({f_{m}}^{2}-f^{2}\right)}^{2}+{\left(f\cdot \Gamma_{m}/Q_{m}\right)}^{2}},$$

where $C^{\prime }$ is a frequency-independent constant encompassing the detector noise, optomechanical coupling, and readout gain. Equation (12) reveals two distinct operating regimes. For resonant devices biased near $f_{m}$, NEP follows a Lorentzian-like response and reaches a minimum at resonance, with an effective bandwidth of approximately $f_{m}/Q_{m}$. For broadband devices operated well below $f_{m}$, the frequency-dependent term is negligible and NEP remains approximately flat across the working band.

This result reflects the fundamental constraint imposed by mechanical thermal noise on the detection limit. In practice, the detection limit is also bounded by laser frequency noise, shot noise, detector and electronic noise, and environmental acoustic noise.

When evaluating acoustic sensing performance, it is important to distinguish the optical quality factor $Q_{0}$ from the mechanical quality factor $Q_{m}$, as they govern different aspects of device performance. The optical quality factor $Q_{0}=\omega_{0}/\kappa$ determines the slope of the resonance transmission curve, ∂T/∂Δ, which scales approximately linearly with $Q_{0}$ and thus governs the efficiency of converting frequency modulation into intensity variation. A higher $Q_{0}$ can therefore improve the optical conversion efficiency under otherwise identical conditions; however, it also narrows the optical linewidth and increases susceptibility to laser frequency noise and environmental drift, so the net sensitivity improvement is not guaranteed and depends on the dominant noise source.

In contrast, the acoustic bandwidth is not directly set by $Q_{0}$. It is primarily determined by the mechanical transfer function $\chi_{m}\left(f\right)$, the mechanical damping $\Gamma_{m}$, the acoustic coupling scheme, the sensor geometry, and the detection method. For mechanical resonance-enhanced devices, the effective bandwidth is approximately $\Delta f\approx f_{m}/Q_{m}$, where $Q_{m}=\Omega_{m}/\Gamma_{m}$ is the mechanical quality factor. The sensitivity–bandwidth trade-off can be quantified from Equation (12): at resonance, the minimum NEP scales as $NEP_{min}\propto {f_{m}}^{2}/Q_{m}$, while the bandwidth scales as $\Delta f\propto f_{m}/Q_{m}$; consequently, their product $NEP_{min}\cdot \Delta f$ is approximately independent of $Q_{m}$. Thus, increasing $Q_{m}$ lowers the NEP near resonance but proportionally narrows the bandwidth, and broadband designs must deliberately reduce $Q_{m}$ or operate below resonance to maintain a flat response over a wider frequency range.

The readout bandwidth, set by the laser control, photodetector, and back-end electronics, imposes an additional upper bound. In practice, thermal drift, coupling-gap drift, and laser-locking stability also affect the operating point stability and long-term detection performance.

In summary, the sensing mechanisms of WGM optical microcavity acoustic sensing can be grouped into dispersive coupling and dissipative coupling. The detection performance of practical devices is jointly determined by the mechanical response, optical conversion efficiency, operating point state, and various noise sources. These mechanisms and performance fundamentals provide theoretical support for the design and performance evaluation of WGM optical microcavity acoustic sensors.

## 4. Medium Adaptation and Function Extension of WGM Optical Microcavity Acoustic Sensing

Building on the mechanisms and performance fundamentals established in Section 3, this section shifts focus to medium adaptation and function extension. Based on the propagation medium and application objectives, representative studies are organized into four categories: air-coupled acoustic sensing, acoustic sensing in liquid environments, photoacoustic imaging, and multifunctional sensing. The first two categories address medium adaptation. Identical opto-acoustic mechanisms face distinct acoustic impedance and damping constraints in air versus liquid environments, requiring scenario-specific device designs and packaging strategies. The latter two reflect function extension. Acoustic detection progressively evolves toward imaging modalities and multiparameter cooperative measurement, as exemplified by photoacoustic imaging and multifunctional sensing. Key device parameters of the representative studies are summarized in the corresponding tables presented in the following subsections.

### 4.1. Acoustic Sensing in Different Propagation Media

#### 4.1.1. Air-Coupled Acoustic Sensing

WGM optical microcavities exhibit unique advantages in air-coupled acoustic sensing, making them an ideal platform for high-sensitivity acoustic detection. However, a severe acoustic impedance mismatch exists at the air–solid interface, causing most of the acoustic energy to be reflected. Meanwhile, ultrasound suffers considerable attenuation in air, and the absorption loss at high frequencies is approximately proportional to the square of the frequency. Consequently, achieving high sensitivity has become the central goal of research on air-coupled acoustic sensing.

**Figure 4:**
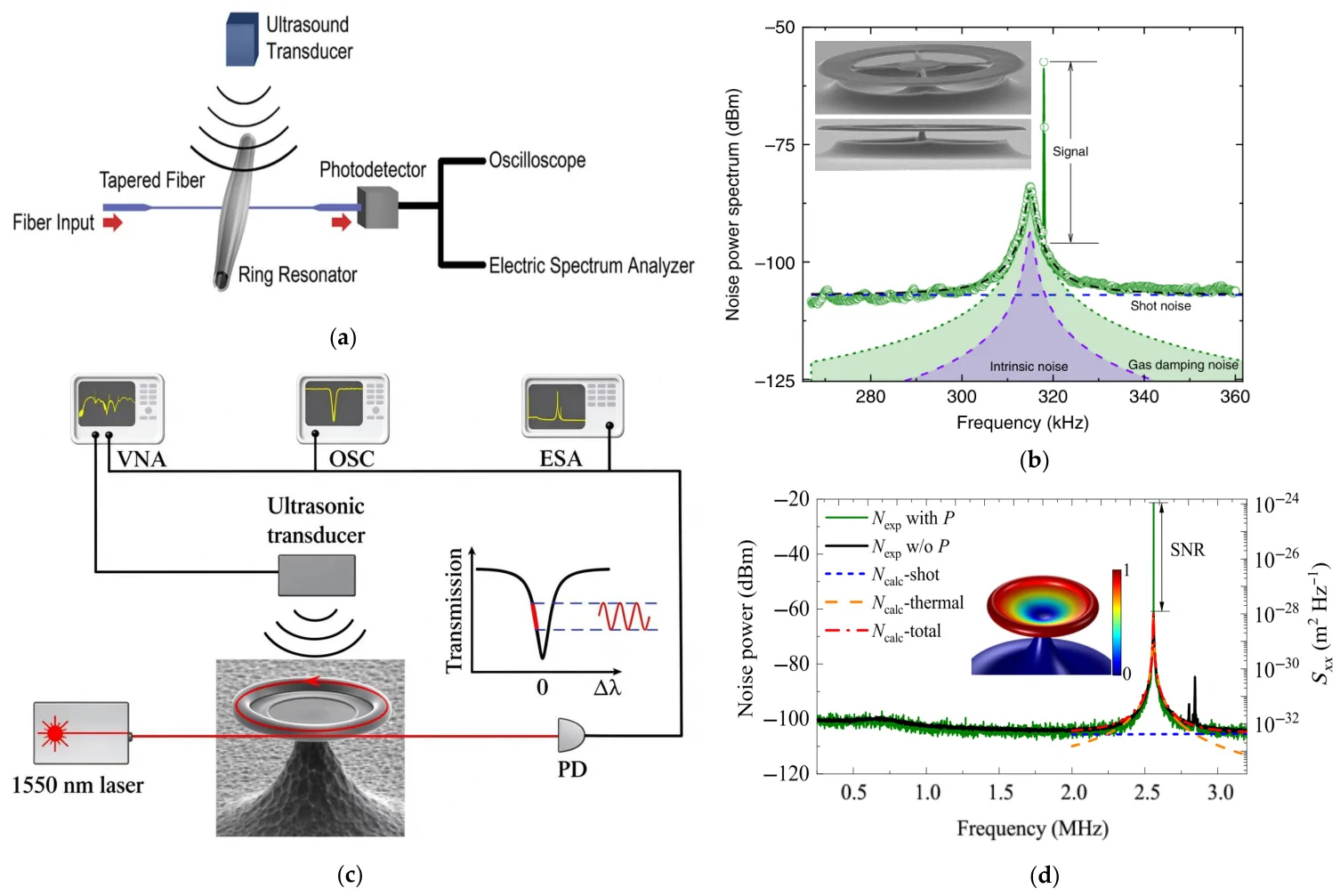
(**a**) A schematic of the experimental setup for ultrasound detection based on a hollow-capillary microbubble cavity; the arrows indicate the propagation direction of the optical signal [126]; (**b**) a noise power spectrum of the sensor near the microdisk mechanical vibration mode; the green curve denotes the signal, the blue dashed line denotes shot noise, the purple dashed line denotes intrinsic noise, and the green dashed line marks the gas damping noise region. Inset: Scanning electron micrograph of a suspended spoke silicon microdisk [129]. (**c**) A schematic of the experimental setup for microtoroid cavity ultrasonic sensing; the arrows indicate the propagation direction of the optical signal [16]; (**d**) a noise power spectrum under ultrasonic excitation and the response curve of the microtoroid cavity. Inset: Normalized simulated displacement distribution of the first-order flapping mode [16].

To date, existing research can be broadly divided into two types according to the different acoustic sensing mechanisms involved: dispersive coupling and dissipative coupling. Among these, dispersive coupling is the most widely adopted acoustic sensing mechanism, in which the mechanical response can be enhanced through structural optimization to approach the thermal noise limit. In 2017, Kim et al. [126] were the first to apply a hollow-capillary microbubble cavity for non-contact detection of air-coupled ultrasound (Figure 4a). The measured noise-equivalent pressures (NEPs) at 50 kHz and 800 kHz were 215 mPa/Hz^1/2^ and 41 mPa/Hz^1/2^, respectively, providing an initial validation of the concept’s feasibility. This work demonstrated the basic feasibility of hollow-cavity-based air-coupled ultrasound detection; nevertheless, its operating bandwidth was limited by intrinsic mechanical resonances, and subsequent works optimized mechanical structures to balance sensitivity and bandwidth. To enhance sensitivity, in 2019, Basiri-Esfahani et al. [129] constructed a cavity optomechanical ultrasonic sensing system by coupling a suspended spoke silicon microdisk with a tapered optical fiber (Figure 4b). They enhanced the device’s mechanical compliance to effectively reduce mechanical damping, thereby achieving thermal noise-limited detection for the first time at room temperature and atmospheric pressure, with an NEP as low as 8–300 μPa/Hz^1/2^@1 kHz–1 MHz. Although this device achieved exceptionally high sensitivity, its optimal response occurred only at discrete mechanical resonance frequencies, limiting its effective operating bandwidth.

While thermal noise-limited sensitivity was achieved, the high-performance response was restricted to discrete resonant points. Researchers further modified substrate-induced mechanical constraints to extend the usable frequency range. To address the limited frequency range of air-coupled ultrasound, in 2022, Yang et al. [16] employed a two-step etching process to fabricate a microtoroid cavity with an ultra-thin silicon substrate (Figure 4c). By reducing the mechanical constraint imposed by the substrate, they achieved an optical *Q* factor of approximately 10^7^ and a mechanical *Q* factor of approximately 700. This was the first time that the frequency range of air-coupled, high-sensitivity ultrasonic detection had been extended to the megahertz range. Within a 0.6 MHz range around the 2.56 MHz mechanical resonance, they achieved thermal noise-limited sensitivity, with an NEP as low as 46 μPa/Hz^1/2^–10 mPa/Hz^1/2^ at 0.25–3.2 MHz (Figure 4d). The following year, they optimized the geometric parameters of the microdisk and introduced etched-trench structures to increase the pressure difference and enhance the mechanical response, achieving a peak NEP of 1.18 μPa/Hz^1/2^@82.6 kHz in air [17]. To the best of the authors’ knowledge at the time of publication, this value represented a record-low NEP among cavity-optomechanical ultrasonic sensors at this specific operating frequency. Note that this performance is constrained by its experimental conditions, including operating frequency, fiber-taper readout scheme and coupling configuration. Detailed parameter comparison is shown in Table 3. The above studies demonstrate that mechanical resonance enhancement can effectively improve sensing sensitivity, but they also reveal the inherent trade-off between high sensitivity and operating bandwidth in resonant sensors.

In addition to optimizing the mechanical structure, improving the detection scheme is also an important route to enhancing the performance of dispersive coupling acoustic sensors. In 2020, Pan et al. [18] combined a high-*Q* microbubble resonator (MBR) with a digital optical frequency comb (DOFC), achieving an NEP of 4.4 mPa/Hz^1/2^@165 kHz. Compared with the laser frequency tuning method, the DOFC method can capture the complete resonant mode spectrum in a single shot on the microsecond scale, featuring a faster response speed, fm-level resolution, and superior noise robustness. Also in 2020, Song et al. [19] combined a high-*Q* chalcogenide glass microdisk resonator with DOFC technology, achieving a detection sensitivity of 2.86 dB/Pa at 40 kHz. They also demonstrated arrayed microcavity detection using three cascaded SiN_x_ microring resonators (MRRs), enabling acoustic source localization in three-dimensional space. Also in 2020, Yang et al. [20] proposed a selectively etched add-drop MRR structure based on a complementary metal oxide semiconductor (CMOS)-compatible silicon-on-insulator (SOI) platform. By etching the target region to enhance the ultrasonic deformation response while retaining the coupling region to prevent deformation-induced changes in the coupling coefficient, they achieved a linear, distortion-free sensing response to air-coupled ultrasonic signals. In 2021, Li et al. [21] employed a cantilever–microsphere cavity structure to achieve highly sensitive, high-fidelity acquisition and reconstruction of speech signals at a distance of 7 m in air, extending the application of WGM optical microcavities to the audible frequency range. In 2022, Meng et al. [22] proposed a thermally assisted microcavity Raman laser ultrasonic detection scheme. It uses the thermal effect in a high-*Q* microsphere cavity to mitigate low-frequency noise while preserving high-frequency sensitivity. The device is resistant to laser frequency drift and environmental interference, enabling long-term stable operation.

**Figure 5:**
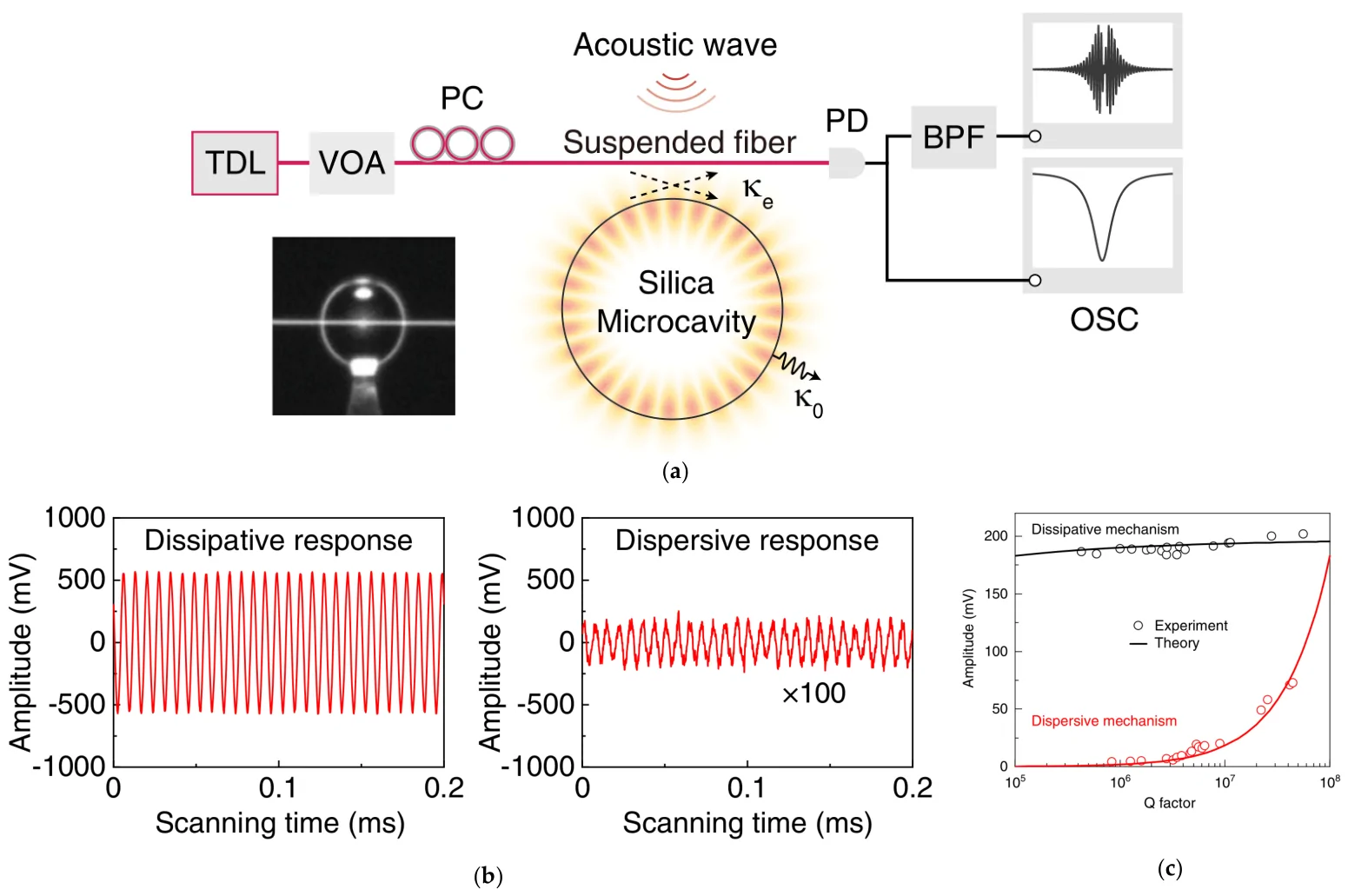
Dissipative acousto-optic responses in a microsphere–microfiber coupled ultrasonic sensor [24]. (**a**) Schematic of strong dissipative acousto-optic interaction between microsphere cavity and suspended vibrating microfiber. Inset: Optical image of microsphere–microfiber coupling system. (**b**) Comparison of ultrasound-induced transmitted light intensity changes under dissipative and dispersive mechanisms; (**c**) detection sensitivity of dissipative and dispersive mechanisms at different *Q* factors.

Unlike the aforementioned dispersive coupling schemes that rely on mechanical resonance, in 2022, Meng et al. [24] achieved an NEP of 0.81 Pa@140 kHz in air by exploiting the strong dissipative acousto-optic interaction between a microsphere cavity and a suspended vibrating microfiber (Figure 5a). Compared with the dispersive response in the same device, the dissipative response driven by external acoustic excitation was two orders of magnitude stronger (Figure 5b). This observed strong response under specific coupling conditions shows weak apparent dependence on the microcavity Q factor (Figure 5c) and is free from mechanical resonance constraints, thereby effectively circumventing the inherent bandwidth limitation of resonant sensors. Nevertheless, the actual detection limit of dissipative sensing is still jointly governed by coupling gap conditions, resonant characteristics, laser noise, detector noise and operating point selection; weak apparent Q-independence only holds for this particular experimental configuration and cannot be treated as a universal physical conclusion. Although the dissipative coupling mechanism can achieve a broadband response, its overall sensitivity remains inferior to that of high-performance resonant dispersive coupling devices, and the sensor operating point is highly sensitive to the waveguide–microcavity coupling gap.

**Figure 6:**
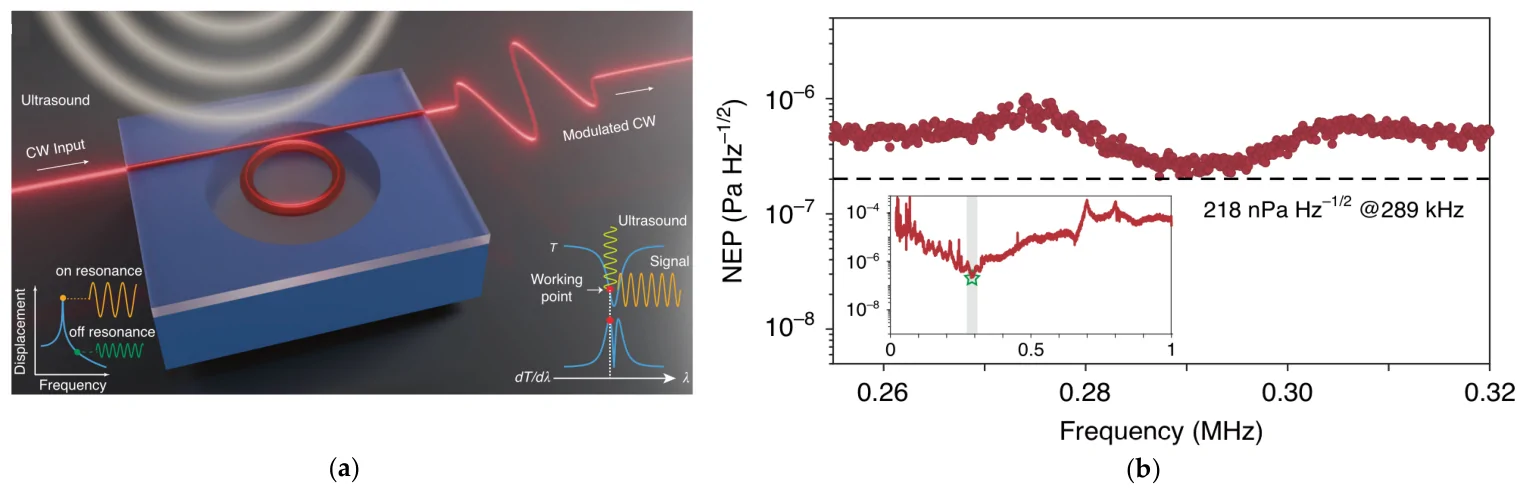
(**a**) A schematic of the integrated ultra-high-sensitivity optomechanical ultrasonic sensor. Left inset: Mechanical resonance-enhanced displacement response. Right inset: Optical readout principle of the sensor [26]. (**b**) The NEP spectrum near the first-order flapping mode in air. Inset: The NEP spectrum in air over 20 kHz–1 MHz; the grey shaded region in the inset is magnified and shown in the main panel, corresponding to the first-order flapping mode ν(0,0), and the star (pentagram) marks the best sensitivity (218 nPa/Hz^1/2^ at 289 kHz) [26].

As sensing mechanisms continue to be explored in depth, device architectures have also evolved toward miniaturization and integration. In 2024, Huang et al. [25] adopted a glass-blowing process to fabricate on-chip microsphere shell resonators, achieving an NEP of 40 μPa/Hz^1/2^@1.72 MHz over 0.6–3.5 MHz. The mechanical vibration modes were cross-validated through three-dimensional finite-element simulations and measurements with a laser Doppler vibrometer. In 2026, Cao et al. [26] developed a CMOS-compatible integrated optomechanical ultrasonic sensor based on a suspended SiO_2_ membrane structure embedding a high-Q Si_3_N_4_ MRR (Figure 6a). It achieved an NEP of 218 nPa/Hz^1/2^@289 kHz in air (Figure 6b). At the time of publication, this value represented a record-low NEP among microcavity-based air-coupled ultrasonic sensors at this operating frequency. Note that this performance is constrained by its specific experimental conditions including operating frequency, readout scheme and packaging configuration. Detailed parameter comparison is shown in Table 3.

In brief, substantial progress has been achieved for air-coupled acoustic sensing using WGM optical microcavities. Resonant sensors built upon dispersive coupling can reach ultra-low noise-equivalent pressure near mechanical resonant frequencies, yet their usable bandwidth is inherently bounded by mechanical-mode responses. By contrast, dissipative-coupling schemes enable broadband acoustic response, albeit with compromised absolute sensitivity. Severe acoustic impedance mismatch at the air-solid interface remains a fundamental physical limitation. In addition, operating point drift and the absence of standardized calibration protocols further complicate quantitative performance evaluation for air-coupled sensor prototypes.

#### 4.1.2. Acoustic Sensing in Liquid Environments

Underwater and general liquid-phase environments impose stringent requirements on acoustic sensors, including waterproofing, pressure resistance, and long-term stability, making packaging design a critical factor that limits their practical application. Unlike air-coupled acoustic sensing, the core challenge for liquid environment acoustic sensing lies in enabling highly sensitive WGM optical microcavities to maintain long-term stable operation in liquid environments. A well-designed packaging structure must not only ensure the optical performance of the microcavity in underwater environments but also simultaneously achieve high acoustic transmittance and mechanical robustness.

**Figure 7:**
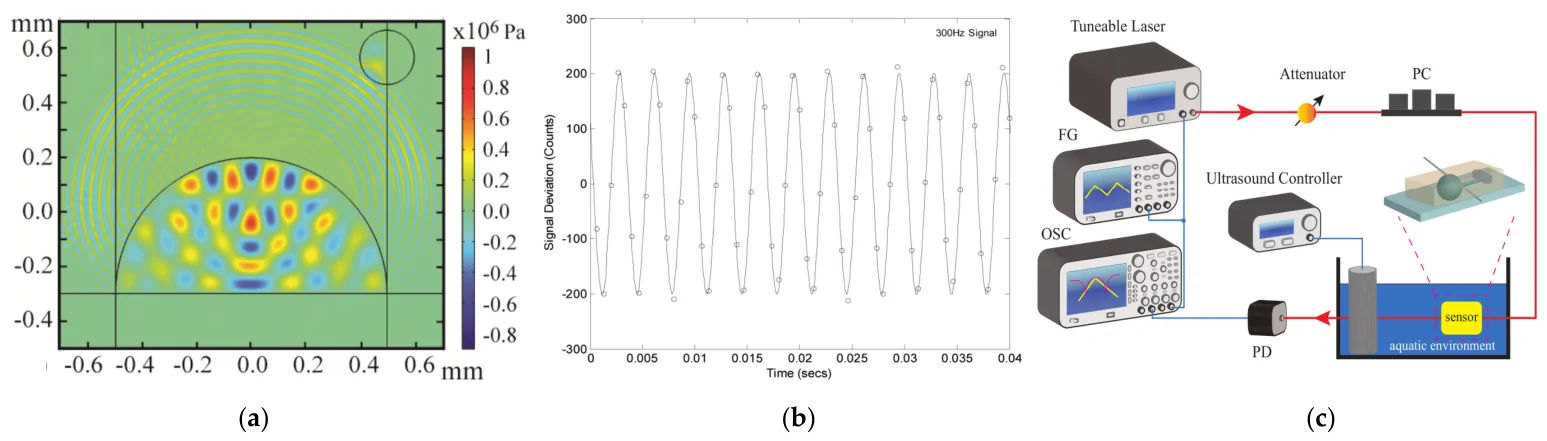
(**a**) FEM simulation result showing the ultrasound echo from the steel sphere reaching the microsphere surface [131]; (**b**) the underwater acoustic response of the micro-knot hydrophone at 300 Hz [132]; (**c**) the experimental scheme of ultrasound detection [135].

Researchers have verified the basic feasibility of various encapsulation schemes in liquid environments. In 2013, Monifi et al. [130] were the first to apply polymer-encapsulated SiO_2_ microtoroid resonators in liquid environments for ultrasonic detection. The *Q* factor of the encapsulated device remained stable, achieving a response sensitivity of 35 mV/kPa at 12 MHz, and it operated stably in complex liquids such as physiological saline and serum. This pioneering work validated that proper packaging enables WGM microcavity operation in liquid media; multiple combinations of cavity geometry and packaging materials were subsequently explored. In 2014, Chistiakova et al. [131] utilized an encapsulated high-*Q* SiO_2_ microsphere cavity for underwater ultrasonic sensing. They demonstrated through COMSOL simulations and experimental validation that this structure could detect echo signals from a steel ball and a water tank (Figure 7a). In 2015, De Freitas et al. [132] first proposed an underwater acoustic sensor based on a silicone rubber-encapsulated optical microknot resonator, with a normalized sensitivity of −288 dB re 1 V/μPa, equivalent to a sensitivity of 5.83 fm/Pa (Figure 7b). In 2019, Chen et al. [133] proposed a high-frequency hydrophone based on a fiber microknot resonator, capable of performing underwater high-frequency acoustic measurements in the several megahertz range. In 2020, Huang et al. [134] utilized a microtoroid optomechanical resonator to achieve high-sensitivity detection of underwater acoustic signals. In addition, they achieved direct frequency down-conversion in self-oscillation mode, enabling the extraction of baseband signals without an external local oscillator. In 2022, Wang et al. [135] utilized an encapsulated microsphere cavity coupled with a tapered optical fiber to achieve underwater ultrasonic sensing (Figure 7c), with the *Q* factor of the encapsulated device maintained at approximately 10^6^. It offers advantages including low cost, ease of fabrication, and reusability. The above studies confirm that, with appropriate encapsulation techniques, WGM optical microcavities are capable of high-sensitivity acoustic detection in liquid environments. Most early liquid environment demonstrations targeted high-frequency ultrasound. For hydroacoustic applications requiring a low-frequency response, broadband packaged microcavity schemes have become an important research direction.

After the basic feasibility of the encapsulation scheme had been verified, the researchers further focused their studies on broadening the detection bandwidth and improving environmental adaptability. In 2022, Tu et al. [136] were the first to propose an underwater acoustic sensor based on an encapsulated MBR, achieving broadband detection from 10 Hz to 100 kHz. They measured an NEP of 2.2 mPa/Hz^1/2^ at 1 kHz, and the sensitivity remained virtually unaffected by variations in hydrostatic pressure and temperature. The −6 dB beamwidths in two orthogonal directions were measured to be 75.6° and 105.5°, respectively. This demonstrates that the sensor possesses favorable environmental stability and relatively broad directivity. This indicates that the encapsulated WGM optical microcavity can not only achieve broadband detection but also exhibit preliminary environmental robustness. While broadband performance was realized, low-frequency acoustic detection still suffers from signal shielding induced by packaging layers, which becomes a major bottleneck for further sensitivity promotion.

Following the above research on broadband detection, low-frequency underwater acoustic detection has gradually become a key focus. However, the encapsulation layer can shield low-frequency sound waves, which has become a major challenge limiting improvements in low-frequency detection sensitivity. In 2023, Chu et al. [137] proposed, for the first time, a highly sensitive low-frequency fiber-optic hydrophone based on a high-*Q* (1.78 × 10^7^) SiO_2_ optical waveguide resonator. The device achieved a flat response of ±2 dB from 40 to 1250 Hz, with a sensitivity of −163.1 dB re 1 V/μPa at 1 kHz, which exceeds that of a standard hydrophone by 33.9 dB. The system used Pound–Drever–Hall (PDH) frequency-locking technology to demodulate underwater acoustic signals, achieving a frequency resolution as low as 1 Hz and a minimum measurable acoustic pressure of 50 mPa/Hz^1/2^. This work provides a new highly sensitive sensing approach for low-frequency underwater acoustic detection.

**Figure 8:**
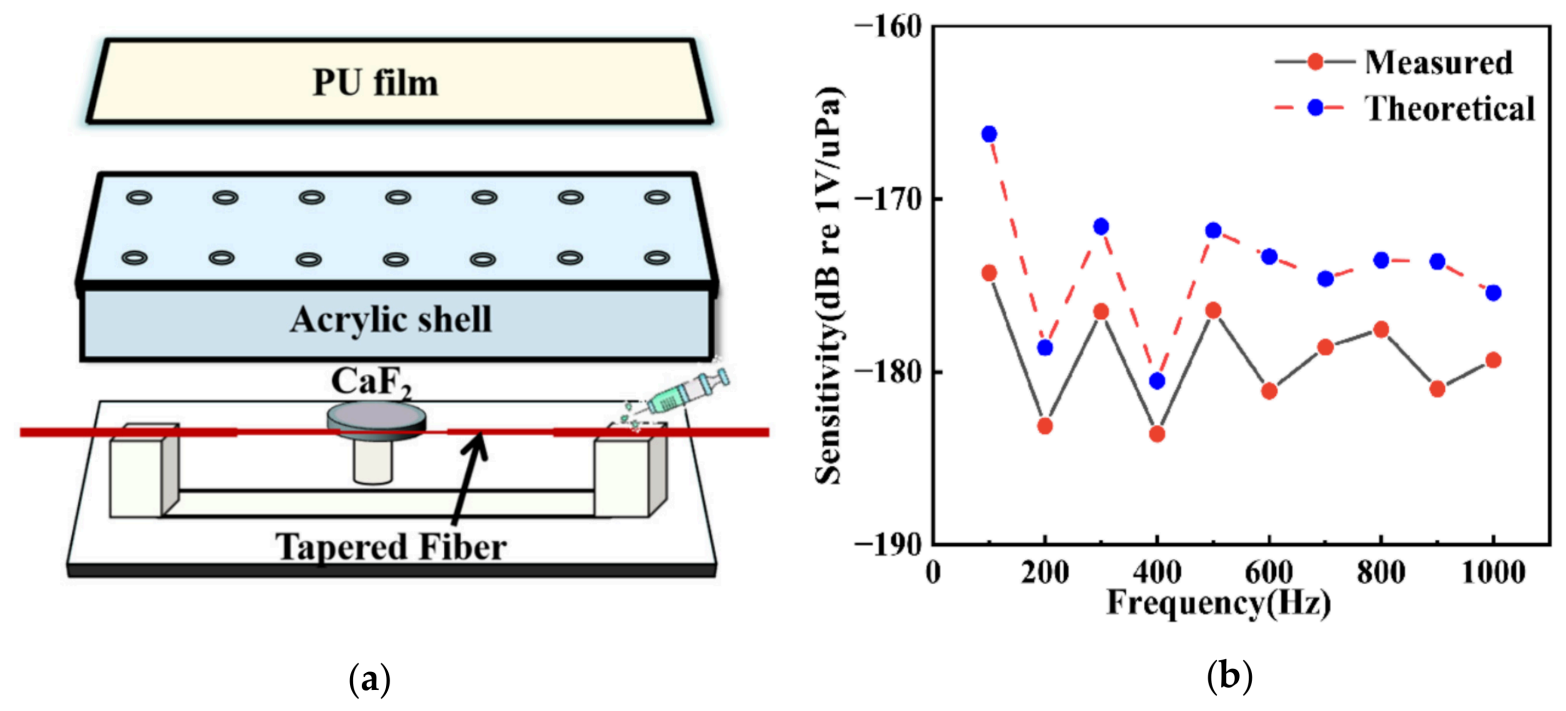
(**a**) A schematic of the perforated array encapsulation for the acoustic sensor based on a CaF_2_ microdisk resonator [125]. (**b**) Comparison of the theoretical and experimental sensitivities of the CaF_2_ microdisk resonator [125].

To address the shielding of low-frequency acoustic signals by the encapsulation layer, in 2024, Yuan et al. [125] employed a high-*Q* (1.2 × 10^8^) CaF_2_ optical resonator for low-frequency underwater acoustic sensing. They adopted a novel hollowed-out array encapsulation structure (Figure 8a), which increased acoustic transmittance by 30 dB compared with flat-panel encapsulation and effectively mitigated the shielding of low-frequency acoustic signals by the encapsulation layer. The bandwidth-fitted acoustic pressure sensitivity was 0.92 mV/Pa over the 100 Hz–1 kHz range, while the single-frequency calibrated sensitivity at 300 Hz reached −176.3 dB re 1 V/μPa (Figure 8b). All above-mentioned experiments were implemented under well-controlled laboratory conditions. Field-level validation under real marine environments is essential for practical deployment.

**Figure 9:**
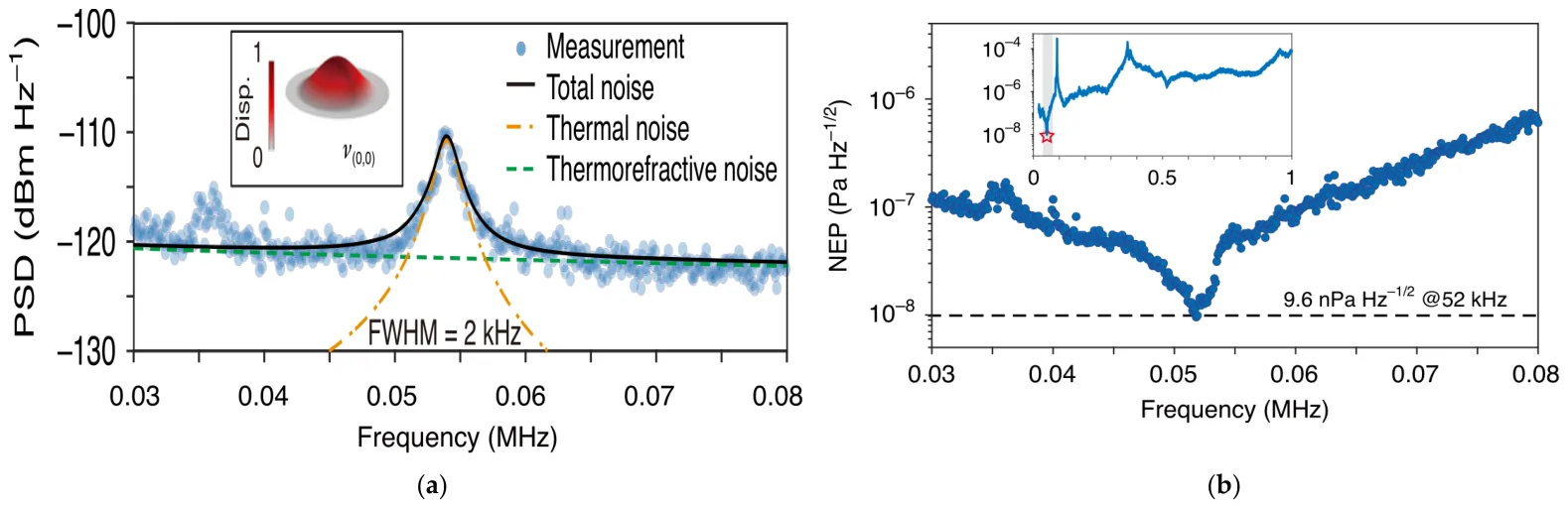
(**a**) Noise power spectral density (PSD) of the first-order flapping mode in water. Inset: the mode profile of the SiO_2_ membrane [26]; (**b**) the NEP spectrum near the first-order flapping mode in water. Inset: NEP spectrum in water over 20 kHz–1 MHz; the grey shaded region in the inset is magnified and shown in the main panel, corresponding to the first-order flapping mode ν(0,0), and the star (pentagram) marks the best sensitivity (9.6 nPa/Hz^1/2^ at 52 kHz) [26].

While metrics such as sensitivity and bandwidth continue to achieve breakthroughs, the engineering practicality and environmental robustness of acoustic sensors have begun to emerge as important aspects in the performance evaluation framework. In 2026, Cao et al. [26] achieved an NEP as low as 9.6 nPa/Hz^1/2^ @52 kHz in water using the aforementioned integrated optomechanical acoustic sensor (Figure 9a,b). This demonstrates nPa-level detection capability; to the best of the authors’ knowledge at the time of publication, this corresponds to a record-low NEP among microcavity-based ultrasonic sensors at this operating frequency under aqueous conditions. Note that optimal sensitivity is only attained near mechanical resonant modes, and performance degrades under full-band operation and practical field-use conditions. Detailed device parameter comparison is provided in Table 4. In the same year, Tang et al. [121] employed a polymer-encapsulated microtoroid cavity with a tapered optical fiber coupling structure, clarifying that its sensing mechanism is primarily governed by the mechanical vibration of the coupled optical fiber over the 100 Hz–100 kHz range, and therefore belongs to the dissipative sensing mechanism. The sensor achieved a sensitivity of −156.95 dB re 1 V/μPa, which is 1–2 orders of magnitude higher than that of a reference hydrophone. In situ lake and ocean trials further verified its good engineering stability and practicality. This marks an important step toward the practical deployment of WGM optical microcavity underwater acoustic sensors in real marine environments.

Research on liquid environment acoustic sensing demonstrates that properly encapsulated WGM microcavities are capable of high-sensitivity acoustic detection under aqueous conditions. Multiple packaging strategies have been validated to balance optical-Q preservation and basic mechanical protection. Nevertheless, encapsulation layers inevitably introduce trade-offs among acoustic transmission, optical loss and long-term durability. In particular, low-frequency acoustic signals are susceptible to shielding effects induced by packaging structures. Additional practical concerns include surface contamination, structural relaxation under prolonged immersion, and the scarcity of field-level experimental validations under real-world marine conditions.

Taken together, WGM optical microcavity acoustic sensors exhibit distinct performance characteristics for air-coupled and liquid-phase detection scenarios. Dispersive-coupling-based resonant devices can attain ultra-low noise-equivalent pressure under appropriate conditions, yet their operational bandwidth is constrained by intrinsic mechanical modes. Dissipative coupling configurations are capable of broadband acoustic response, generally at the expense of absolute detection sensitivity. For liquid-phase measurement, protective packaging introduces multi-objective conflicts among acoustic transmission efficiency, preservation of high optical-Q performance and mechanical durability. These medium-dependent constraints, together with the lack of unified calibration standards and operating point drift, constitute major barriers for practical engineering deployment.

### 4.2. Photoacoustic Imaging

Photoacoustic imaging offers the dual advantages of high contrast and deep penetration inherent to optical imaging. It enables minimally invasive analysis of the structural and functional information of biological tissues, making it highly valuable in biomedical applications such as microvascular visualization, early-stage tumor screening, long-term in vivo monitoring, and precise endoscopic diagnosis. Traditional piezoelectric ultrasonic transducers are constrained by their geometric dimensions, limited bandwidth, and susceptibility to electromagnetic interference. Consequently, they find it difficult to meet the demands of high-performance photoacoustic imaging, which requires high precision, miniaturization, and broad bandwidth. By virtue of their unique advantages, including micrometer-scale dimensions, ultra-high detection sensitivity, ultra-wide frequency response, and resistance to electromagnetic interference, WGM optical microcavities are also highly compatible with various photoacoustic imaging systems. They have therefore become a preferred choice for high-performance all-optical photoacoustic detection.

Early research has demonstrated the feasibility and fundamental performance advantages of polymer MRRs for photoacoustic detection [102,103,138,139,140,141,142,143,144,145,146]. A series of foundational studies conducted before 2016 gradually confirmed that MRR devices offer broad frequency response, low noise, wide acceptance angle, and array scalability. Through device miniaturization, transparent structural design, and optimization for imaging system compatibility, the resolution and signal-to-noise ratio of photoacoustic imaging have been continuously improved. Preliminary breakthroughs have been achieved, ranging from phantom imaging and microvascular visualization to multimodal biomedical imaging, laying a solid foundation for the subsequent development of in vivo monitoring and integrated imaging technologies.

**Figure 10:**
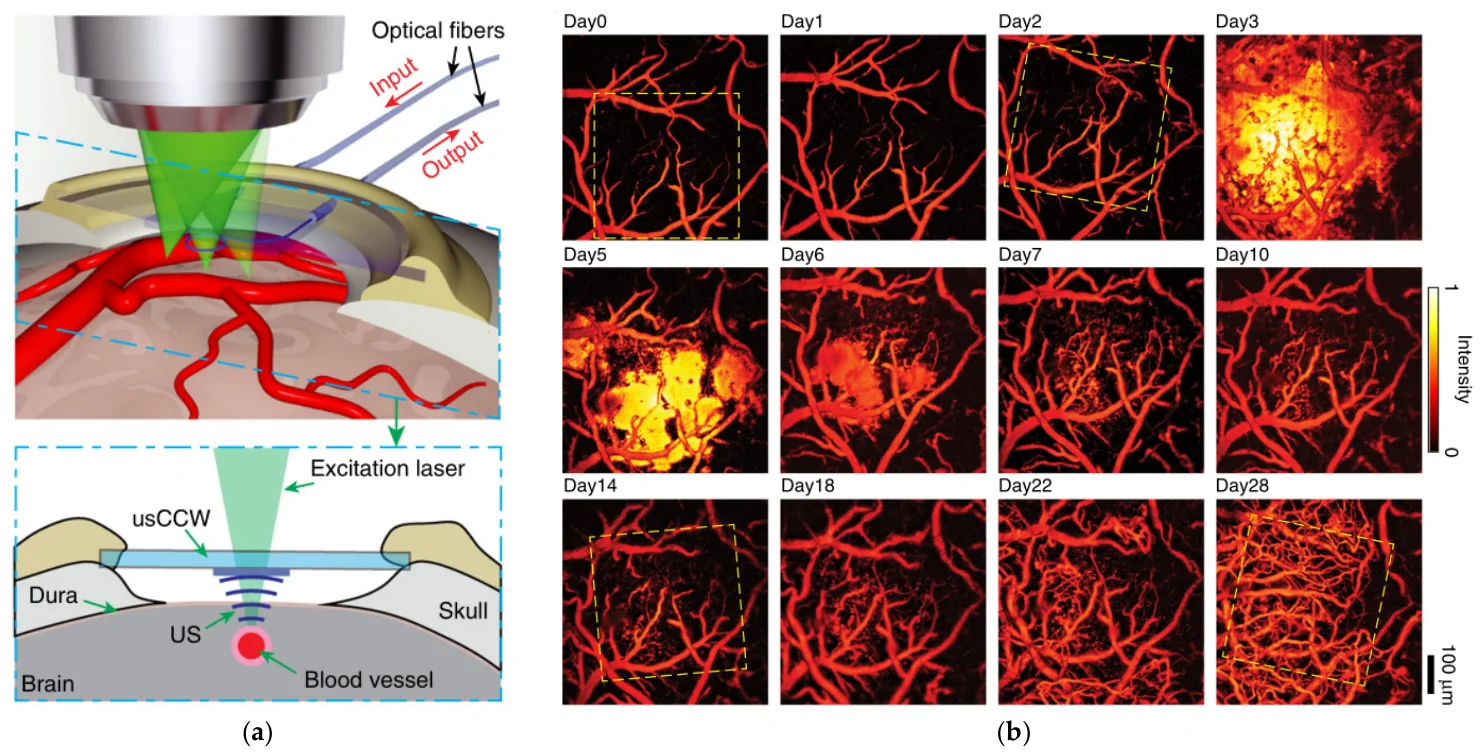
(**a**) Optical scanning process through usCCW. Bottom: Geometry of optical excitation and ultrasound detection shown in highlighted cross-section [147]; (**b**) maximum amplitude projection images of photoacoustic imaging of cerebral cortical vasculature in same region over 28-day period; each image corresponds to one day of the 28-day period [147].

As foundational imaging performance gradually matured, the capability for long-term in vivo monitoring became a crucial step toward the practical application of WGM optical microcavity photoacoustic technology. In 2019, Li et al. [147] developed a disposable ultrasound-sensing chronic cranial window (usCCW) incorporating a transparent nanophotonic ultrasound detector, using soft nanoimprint lithography. After being surgically implanted into the mouse skull, the device established an independent observation system that simultaneously preserved optical pathway and ultrasound detection capability without requiring an external ultrasound coupling medium (Figure 10a). This enabled photoacoustic microscopic monitoring of the vascular network in the cerebral cortex of live mice for up to 28 days (Figure 10b). The axial resolution was approximately 3.57 μm, and the manufacturing cost of a single device was less than one dollar. This provided a low-cost, disposable solution for integrated long-term optical observation and ultrasound sensing in the brain. This study realizes long-term in vivo observation; nevertheless, long-term stability under complex biological fluid environments still needs further verification. Single-channel detectors are limited in imaging throughput and field-of-view; array integration is an essential route to promote practical photoacoustic imaging performance.

**Figure 11:**
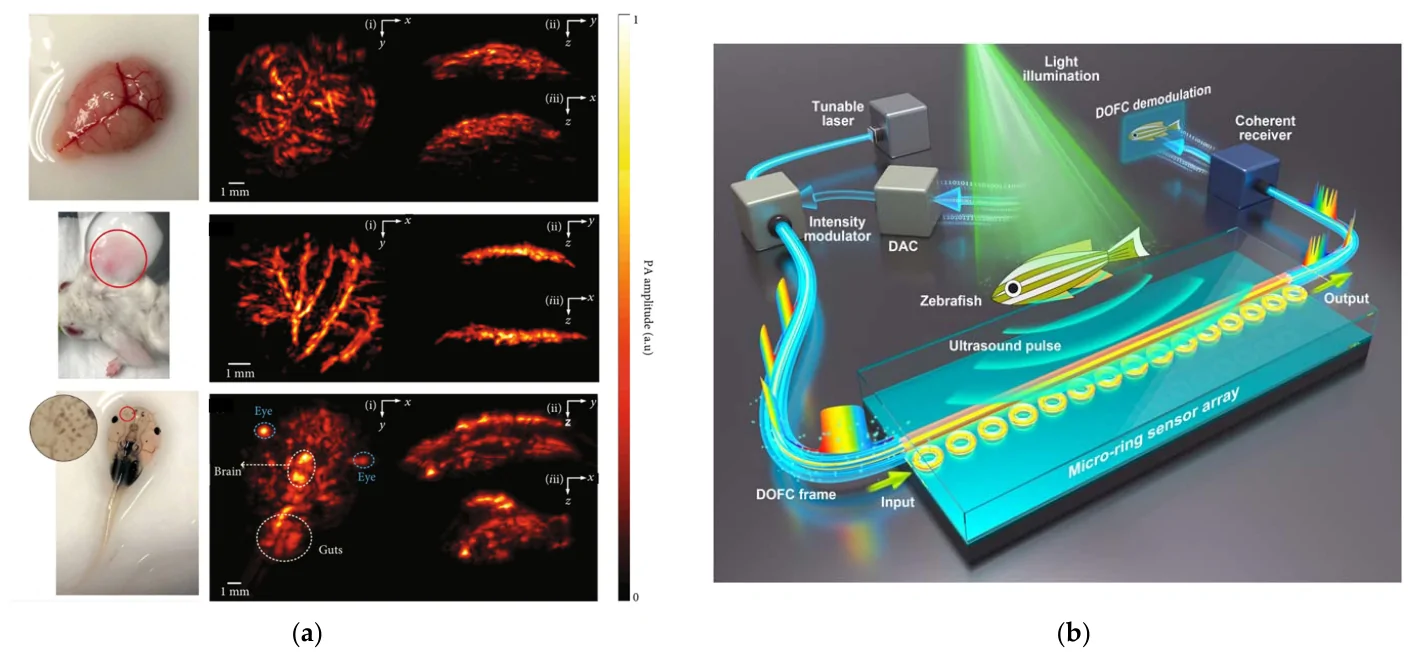
(**a**) 3D-PACT imaging of ex vivo brain tissue and in vivo mouse ear and tadpole; (i)–(iii) denote MAP projections on the xy, yz, and xz planes, respectively; the dashed circles mark the brain, eye and guts regions [104]; (**b**) schematic of PAT using chalcogenide-based microring sensor array [81].

Since 2021, integrated and array-based photoacoustic imaging technologies have achieved significant breakthroughs, effectively addressing the low imaging efficiency and limited field of view of conventional single-channel detection systems. Westerveld et al. [148] proposed an integrated optomechanical ultrasound sensor combining an MRR with a silicon membrane on a CMOS-compatible SOI platform. They used a 15 nm air gap to convert acoustic vibrations into optical signal modulation, achieving an NEP as low as 1.3 mPa/Hz^1/2^ over the 3–30 MHz range. Using a one-dimensional array comprising ten microrings, they verified the feasibility of array detection by measuring the response delays of different microrings. They also successfully performed three-dimensional photoacoustic imaging of a phantom consisting of three overlapping polyamide structures. With the increase in array channel count, multi-channel parallel readout becomes a new bottleneck restricting array practicability. In 2022, Rong et al. [104] integrated an MRR onto a transparent quartz coverslip. With a high *Q* factor of 4.6 × 10^4^, an NEP of 81 Pa, and an ultra-wide 90° acceptance angle, they developed a transmission-mode, high-frequency three-dimensional photoacoustic computed tomography (3D-PACT) system specifically for large-field-of-view imaging of deep tissues. The lateral and axial resolutions reached 114 μm and 57 μm, respectively, and its three-dimensional imaging performance was validated through phantom experiments and in vivo imaging of mice and tadpoles (Figure 11a). To address the low demodulation efficiency of arrays, Pan et al. [81] fabricated an MRR array comprising 15 sensing units from chalcogenide compounds in 2023 (Figure 11b). They also developed a DOFC parallel interrogation technique, enabling a simplified and efficient demodulation scheme using only a single optical source and a single detector. With a maximum bandwidth of 175 MHz and an NEP as low as 2.2 mPa/Hz^1/2^, they successfully performed photoacoustic tomography (PAT) images of rapidly moving objects, leaf veins, and live zebrafish. Although DOFC solves multi-channel readout complexity, hardware cost and massive data throughput remain obstacles for large-scale array deployment. Besides on-chip integrated array schemes, fiber-coupled micro-probes offer another technical path for minimally invasive near-field photoacoustic detection.

**Figure 12:**
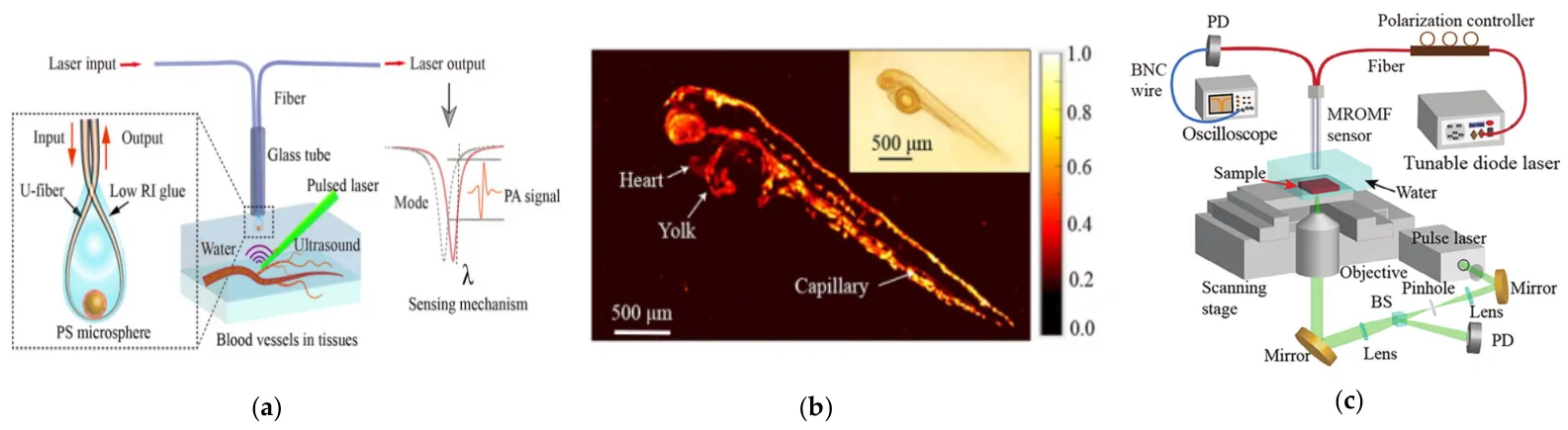
(**a**) A schematic of the working principle of the optical microcavity ultrasound probe. Left inset: Magnified detail of the probe structure. Right inset: The detection mechanism of the ultrasound signal [52]. (**b**) A maximum amplitude projection image of photoacoustic microscopy of a 2-day-old zebrafish. Inset: A bright-field micrograph of the same zebrafish [52] (**c**) The experimental setup of the photoacoustic microscopy system [58].

**Figure 13:**
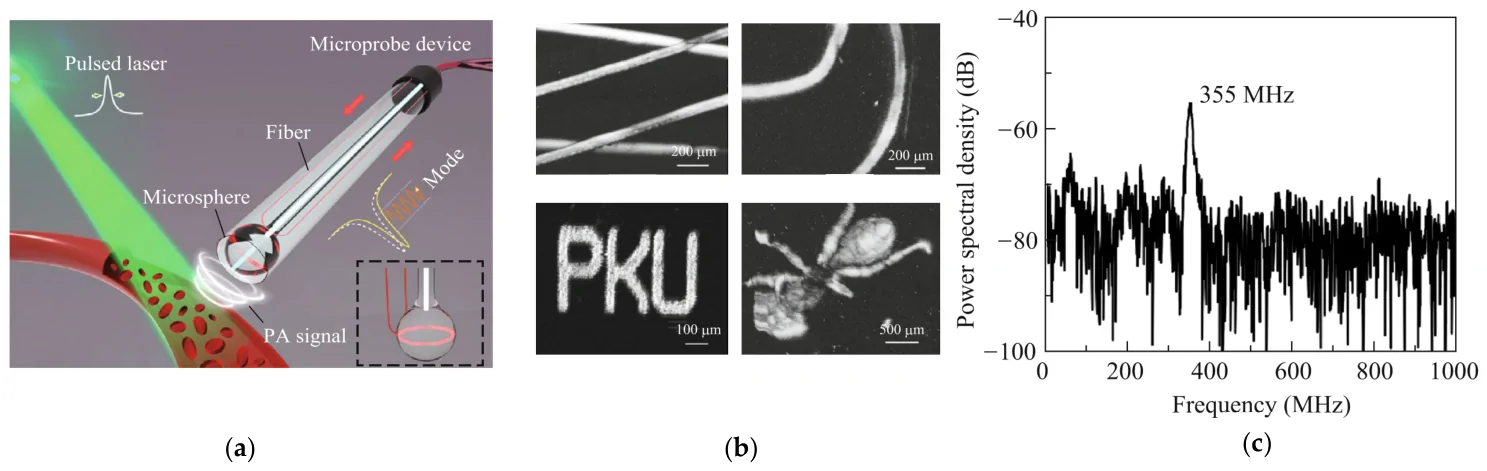
Fully Packaged WGM Microprobe for Photoacoustic Imaging and Vibration Spectroscopy of Micro-Scale Targets [53]. (**a**) A schematic of the microprobe device structure, where the arrows indicate the propagation direction of light inside the microprobe; (**b**) photoacoustic imaging results of straight hair, curly hair, letter-shaped gold thin-film targets, and an ant biological sample; (**c**) frequency-domain vibration signal generated by pulsed-light-excited polystyrene particles.

Meanwhile, miniaturized probe-based photoacoustic detection technology has undergone rapid iteration, gradually advancing from discrete components to fully integrated packaging. The Sun research group has conducted a series of optimization studies on microcavity fiber probes, continuously improving device stability and imaging performance. In 2022, the team proposed a packaged microsphere probe. With a high *Q* factor of approximately 10^6^, it achieved an NEP of 160 Pa@20 MHz and a broad bandwidth of 70 MHz, successfully obtaining three-dimensional photoacoustic images of hair and leaf veins [149]. In 2023, they proposed an optical microprobe consisting of a polymer microsphere and a U-shaped tapered optical fiber (Figure 12a). With an ultra-high *Q* factor exceeding 10^6^, a broadband response of 150 MHz, and an NEP as low as 1.07 mPa/Hz^1/2^, its near-field detection at a distance of only tens of micrometers effectively suppressed ultrasonic attenuation, enabling high-fidelity whole-body photoacoustic imaging of zebrafish larvae (Figure 12b) [52]. In 2024, they were the first to fabricate an ultrasonic detector integrating an MRR with a bridge-coupled waveguide on the end face of a multicore fiber using laser direct writing. Combining the dual advantages of fiber devices and MRRs, the detector achieved an NEP as low as 13.2 mPa/Hz^1/2^ over a broadband response exceeding 250 MHz. They also designed an integrated photoacoustic microscopy imaging scheme with synchronized transmission and reception (Figure 12c), ultimately achieving lateral and axial spatial resolutions of 5 μm and 6.6 μm, respectively, with its key performance parameters listed in Table 5 [58]. In 2025, the team used low-refractive-index polymer microdroplets to couple a folded tapered optical fiber with a SiO_2_ microsphere, reinforced with glass and metal sleeves, thereby fabricating a highly stable, fully packaged WGM microprobe (Figure 13a). It achieved a *Q* factor of 10^7^, an ultrasonic sensitivity of 5.4 mPa/Hz^1/2^, and a bandwidth of 41 MHz. They successfully achieved high-resolution photoacoustic imaging of hair, letter-shaped gold films, and ants (Figure 13b). They simultaneously acquired the vibrational spectral signals of polystyrene particles under pulsed-light excitation (Figure 13c) [53]. Fiber-coupled microprobes offer compact near-field detection and flexible deployment; however, manual fiber–cavity alignment and coupling stability remain practical concerns for array-level expansion. Besides fiber-probe schemes, polymer-based micro-bottle resonators provide another approach for miniaturized photoacoustic sensing with built-in tuning capability.

In 2025, Kuang et al. [69] proposed a low-cost, highly stable polymer microbottle ultrasonic sensing scheme. By integrating an electrothermal tuning structure inside the self-assembled microbottle, they achieved rapid, wide-range resonant frequency sweeping exceeding 33 GHz and optimal mode locking without the need for an expensive tunable laser. The device exhibited a *Q* factor exceeding 10^6^ and an NEP as low as 3.35 mPa/Hz^1/2^. Furthermore, the thermal broadening of the resonant modes enhanced mode-locking stability, which ultimately yielded high-contrast photoacoustic microscopy images with a lateral resolution of 1.6 μm. This scheme lowers the demand for costly tunable lasers via on-chip electrothermal tuning. Even so, its usable acoustic bandwidth may be limited by the intrinsic mechanical characteristics of the polymer, and the long-term thermal performance remains to be further characterized.

Collectively, benefiting from a miniature footprint, broad operational bandwidth and high detection sensitivity, WGM optical microcavities constitute a promising technical foundation for high-performance photoacoustic imaging. Considerable advances have been achieved across multiple research directions, including long-term in vivo monitoring, array-enabled three-dimensional photoacoustic reconstruction, and minimally invasive imaging implemented via fiber-coupled microprobes. Even so, resonant-mode devices are subject to inherent trade-offs between mechanical responsiveness and usable acoustic bandwidth. Further practical obstacles arise from multi-channel crosstalk in sensor arrays and signal stability degradation under complex biological conditions. Such bottlenecks still impede the transition of these imaging modalities from laboratory prototypes toward routine biological practice.

### 4.3. Multifunctional Sensing

In practical applications, there is often a demand for a single sensor to simultaneously measure multiple physical quantities, such as temperature, pressure, refractive index, and flow velocity. Therefore, integrating multiple functions into a single sensor to achieve cooperative detection of multi-physical fields has become an important direction for development. WGM optical microcavities possess inherent advantages in this regard, as their resonant modes are highly sensitive to cavity geometric dimensions, refractive index distribution, and boundary conditions; different modes and readout mechanisms can be used to distinguish disturbances such as temperature, strain, mass loading, and sound pressure. Furthermore, microcavity configurations offer considerable design flexibility, allowing performance to be tailored to different measurands and providing structural freedom for multifunctional integration. However, this also faces significant challenges. Multiple external disturbances can become coupled, causing resonant wavelength shifts and thereby making it difficult to decouple information about individual physical quantities from transmission spectra alone. Trade-offs exist among different sensing functions, and issues such as multi-channel signal crosstalk and the matching of sensitivity and dynamic range must also be addressed, placing high demands on device design and signal processing. Most reported prototypes realize multiparameter responses under isolated, separate experimental conditions. Real-time synchronous decoupling of mutually coupled physical quantities under identical operating environments remains a major bottleneck, which will be further elaborated in Section 5.

Early studies separately verified the ability of a single device to respond to multiple physical quantities. In 2019, Zhang et al. [150] employed a fiber-coupled microsphere resonator and proposed a novel packaging and fixation scheme to enhance its stability. They investigated its response characteristics to bulk refractive index and temperature both theoretically and experimentally. The measured bulk refractive index sensitivity was approximately 20.49 nm/RIU, while the temperature sensitivity was 7.38 pm/°C. In addition, the study applied the microsphere resonator to sound-intensity-level sensing, with a measured sensitivity of approximately −1.9 pm/dB. Thus, refractive index, temperature, and sound intensity were sensed and measured using the same device. The representative structures, materials and functions of such WGM microcavity-based multifunctional sensing devices are summarized in Table 6. Although this device demonstrates multiparameter sensing capability under separate tests, it lacks resonant-enhanced multi-field interaction. Cavity optomechanical effects provide an alternative route for simultaneous multi-physical-signal coupling on a single microstructure.

**Figure 14:**
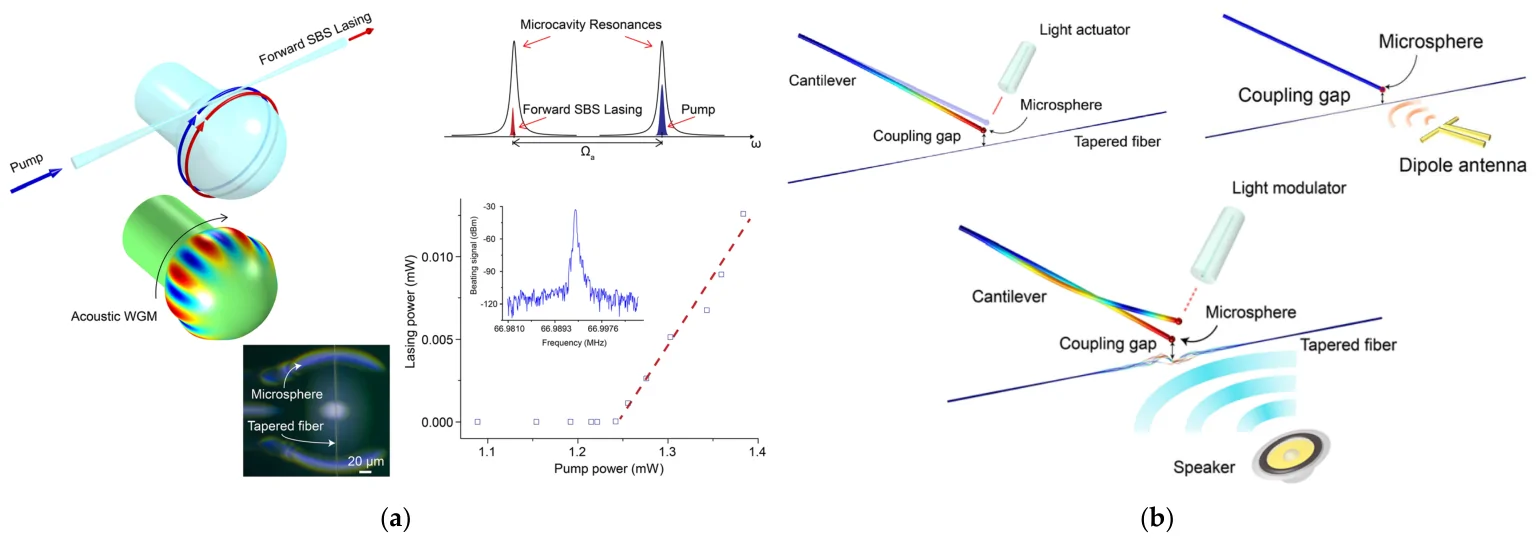
(**a**) A schematic of the working principle of the MBL [151]; (**b**) a schematic of MBL tuning via optical radiation pressure, sound pressure, and microwaves [151].

In 2020, Yang et al. [151] developed a microcavity Brillouin laser (MBL) based on the coupling between a cantilever microsphere and a tapered optical fiber (Figure 14a). By exploiting optical radiation pressure and thermal absorption, they enabled light, sound, and microwaves to interact with the same microstructure in a resonantly enhanced manner at the microscale. Multiphysics sensing of external optical, acoustic, and microwave signals was achieved on a single device, with an acoustic sensing NEP of approximately 267 μPa/Hz^1/2^ (Figure 14b). New applications, including broadband non-photovoltaic optical detection, acousto-optic mixing, and deep-subwavelength microwave imaging, were also demonstrated.

**Figure 15:**
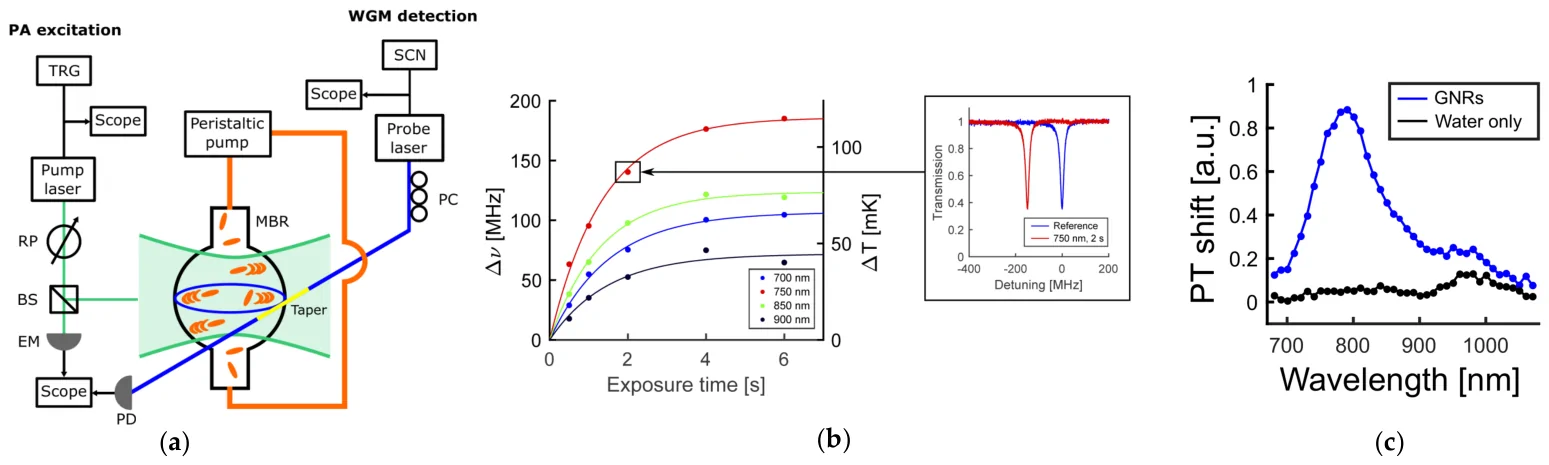
(**a**) A schematic of the experimental setup for photoacoustic signals from plasmonic nanoparticles [153]; (**b**) the resonant optical frequency shift as a function of exposure duration [154]; (**c**) the photothermal spectrum measured after active frequency-locking [156].

Unlike solid microsphere cavities, the hollow structure of MBRs naturally enables them to serve dual roles as both ultrasonic transducers and sample cells. They therefore offer unique potential for multifunctional integration. The Frigenti group has conducted a series of studies using MBRs, systematically exploring their multifunctional integration capabilities in photoacoustic detection and photothermal spectroscopy. In 2019, the group used an MBR, with its microbubble wall serving as an ultrasonic transducer and its internal hollow structure as a sample container, to achieve all-optical photoacoustic detection of submicroliter samples without impedance-matching medium [152]. In 2020, the group further applied MBRs to flow-based photoacoustic detection (Figure 15a). By using the mechanical eigenmodes of the MBR to separate the photoacoustic response from flow noise, they improved the signal-to-noise ratio. They successfully reproduced the photostability curve of a standard contrast agent, making the method suitable for detecting flowing targets such as circulating tumor cells [153]. In 2021, they further exploited the photothermal effect of MBRs. By detecting the resonant wavelength shift caused by nanoparticles absorbing optical energy (Figure 15b), they achieved scattering-free absorption spectroscopy in strongly scattering media [154]. In 2023, the group further integrated photoacoustic detection and photothermal spectroscopy into a single MBR device. By using the MBR’s sharp mechanical eigenmodes to separate the photoacoustic signal from environmental noise, they ensured that the measured spectra were unaffected by sample-scattering effects [155]. In 2024, they introduced active frequency-locking technology to improve system sensitivity by an order of magnitude (Figure 15c), further enhancing the MBR’s performance in combined photoacoustic and photothermal characterization [156]. The performance improvement enabled by active frequency-locking is listed as one controlled-variable test case in Table 7. Even with frequency-locking, cross-interference originating from temperature drift may not be fully suppressed. Optimization limited solely to hardware approaches can struggle to mitigate multi-physical-field crosstalk comprehensively.

**Figure 16:**
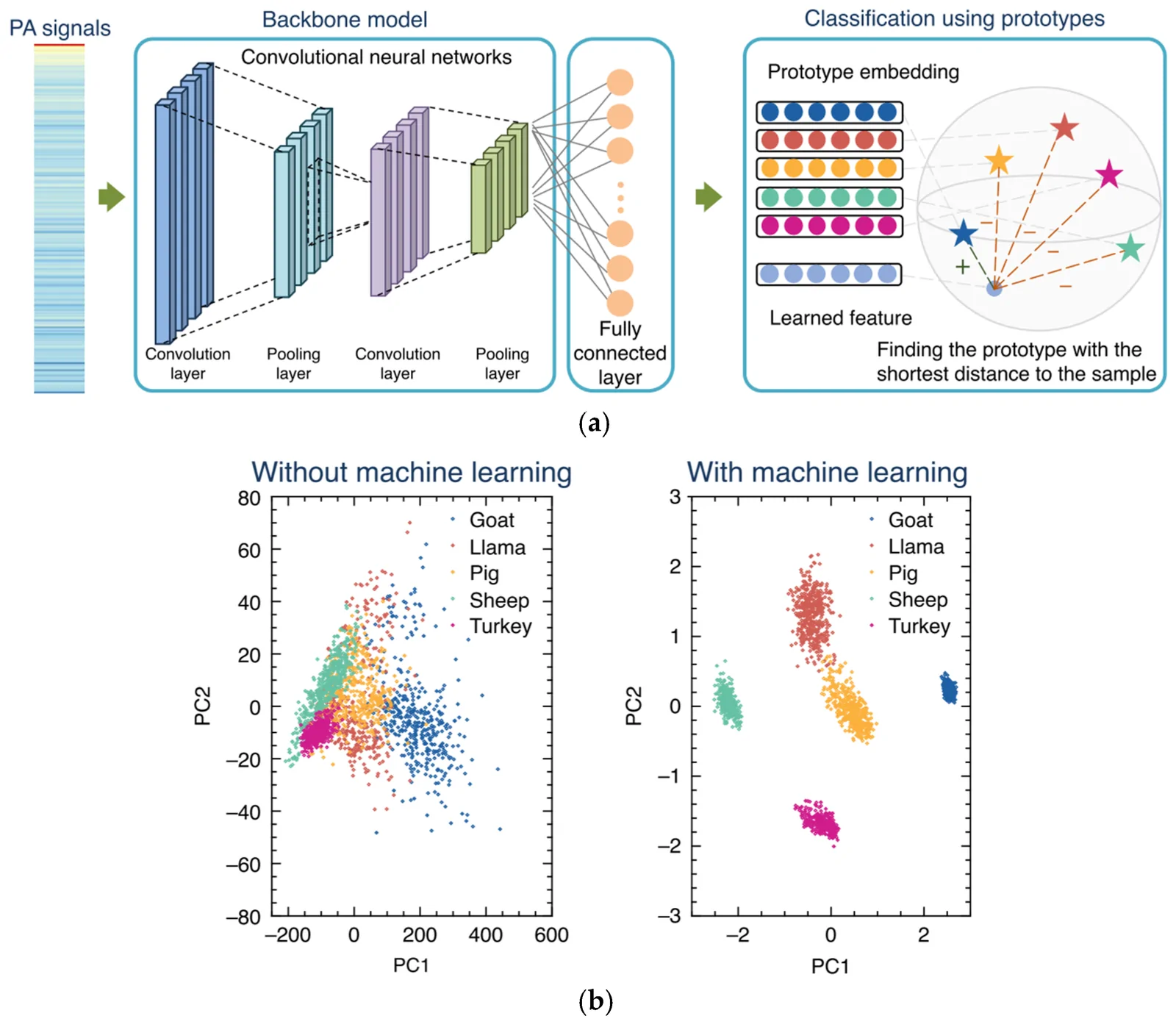
(**a**) An overall schematic of the classification pipeline and a convolutional neural network with a prototype learning structure [80]; (**b**) PCA visualization of samples without machine learning-based feature extraction and of samples using features extracted by the machine learning algorithm [80].

Based on multiparameter detection, researchers have combined WGM optical microcavities with intelligent algorithms, extending their application to the field of target recognition. In 2025, Liao et al. [80] proposed an optofluidic photoacoustic sensor based on an MBR that leverages machine learning algorithms. Within a broad detection range, the sensor enabled high-throughput, label-free detection and classification of freely flowing nanoparticles and cells. By taking advantage of the unique light absorption characteristics of the targets, this technology enabled selective detection in complex samples without the need for surface immobilization. Furthermore, photoacoustic fingerprints combined with machine learning algorithms were used to effectively identify different particles and cells (Figure 16a,b). In addition, the device offers advantages including microfluidic compatibility, superior immunity to optical background interference, and robustness against environmental perturbations such as temperature variations. This work demonstrates machine learning-assisted classification of photoacoustic signals. Beyond algorithm-aided target identification, microcavities with ultra-high Q factors also enable hardware-level, high-precision multiparameter measurement.

**Figure 17:**
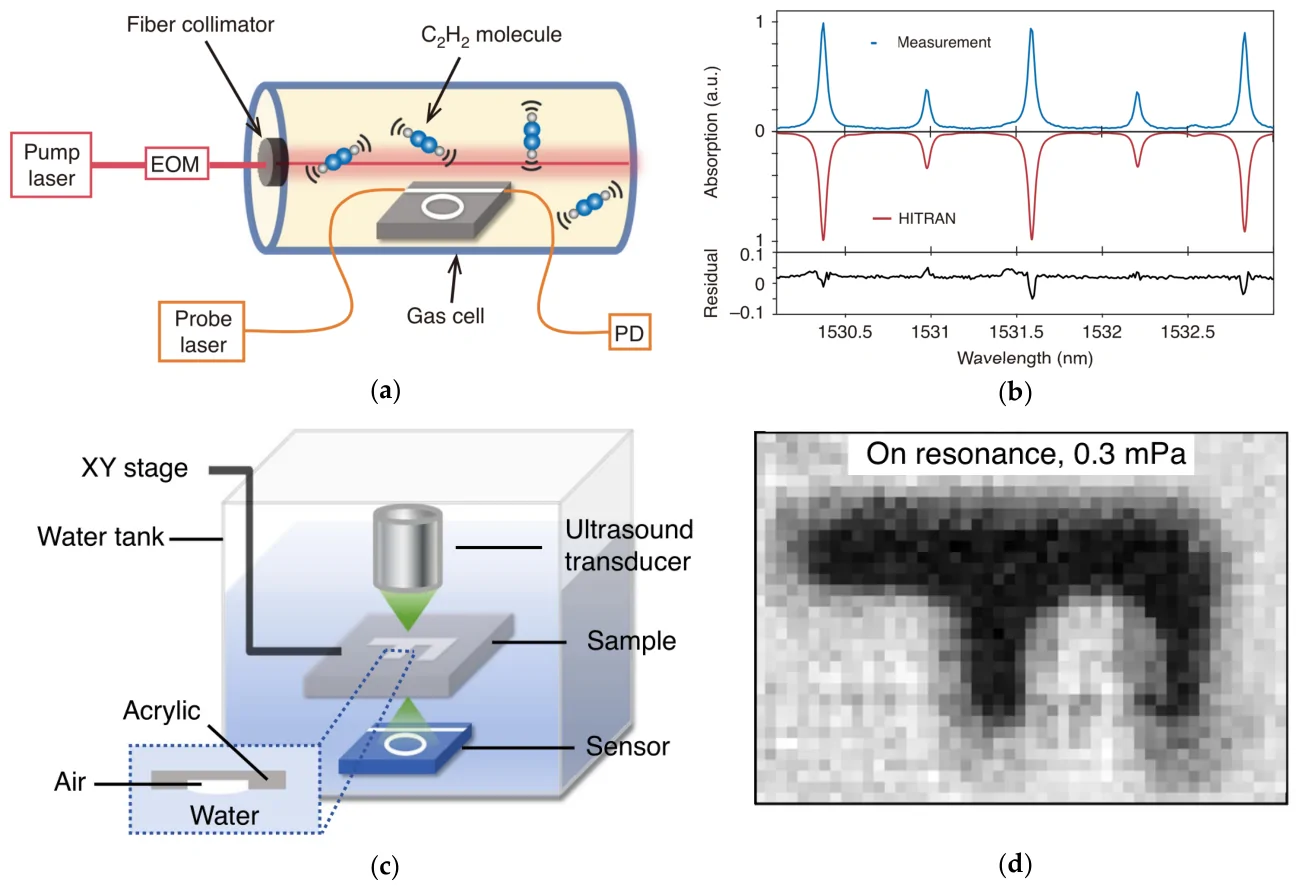
Multifunctional sensing for gas photoacoustic detection and underwater ultrasound imaging [26]. (**a**) Photoacoustic experimental setup for detecting gaseous acetylene; (**b**) a photoacoustic spectrum of acetylene gas; (**c**) a schematic of the underwater ultrasound imaging system; (**d**) underwater ultrasound imaging results.

With the continuous advancement of multi-physical parameter detection and intelligent recognition technology, remarkable breakthroughs have been achieved in high-precision measurement and system integration. In 2024, Pan Ziwen [157] proposed, in his doctoral thesis, a microsphere resonator with a *Q* factor as high as 9.4 × 10^8^. To validate the phonon laser sensing effect in microcavities with ultra-narrow linewidths, three high-precision sensing functions—temperature, mass, and sound pressure—were designed and validated, with resolutions of 4.19 μK, 0.388 fg, and 173.9 μPa, respectively. In terms of integration, in 2026, Cao et al. [26] developed an integrated optomechanical ultrasonic sensor based on the aforementioned integrated optomechanical ultrasonic sensor, combining photoacoustic gas spectroscopy and ultrasonic imaging on a single device. The minimum detectable concentration of acetylene in photoacoustic gas spectroscopy reached 2.9 ppm (with an integration time of 1 s) (Figure 17a,b), while the resolution of ultrasonic imaging reached 1.89 mm (Figure 17c,d). This demonstrated the feasibility of an on-chip multifunctional sensing approach.

**Table 6:** Representative performance of WGM microcavity-based multifunctional sensing devices.

Reference	Device Structure	Material	Functions
[150] (2019)	Microsphere	SiO_2_	Refractive index sensing Temperature sensing Acoustic intensity sensing
[151] (2020)	Cantilever microsphere	SiO_2_	Multi-field sensing (light, sound, microwave) Acousto-optic mixing Microwave subwavelength imaging
[156] (2023)	Microbubble	SiO_2_	Photoacoustic detection Photothermal spectroscopy
[80] (2025)	Microbubble	SiO_2_	Label-free microfluidic photoacoustic detection Particle/blood cell classification
[26] (2026)	Suspended SiO_2_-membrane-embedded Si_3_N_4_ microring resonator	Si_3_N_4_, SiO_2_	Ultrasound detection Photoacoustic gas spectroscopy Underwater ultrasonic imaging

In brief, multifunctional sensing enabled by WGM optical microcavities leverages the multi-physical-field responsiveness of microresonators to expand sensor functionalities beyond standalone acoustic signal acquisition. Various device prototypes have achieved joint measurements of multiple physical quantities, alongside preliminary demonstrations of combined photoacoustic–photothermal characterization and machine learning-aided acoustic signal classification. However, the majority of reported multiparameter responses are validated under well-isolated experimental configurations where physical perturbations are applied independently. Real-time synchronous decoupling of mutually interfering physical quantities under practical operating conditions remains insufficiently demonstrated, representing a key practical constraint for the real-world deployment of multifunctional microcavity sensors.

Comparative analysis of existing sensor performances in Table 3, Table 4, Table 5 and Table 6 reflects overall progress across different application scenarios. To further isolate the influence of individual technical factors on sensing performance, Table 7 summarizes three representative controlled-variable test cases, where only one key parameter is modified while other experimental conditions are kept largely unchanged.

**Table 7:** Performance comparison before and after single-parameter modification for typical WGM microcavity acoustic sensor cases.

Case	Reference	Modified Parameter	Baseline (Before Modification)	Optimized (After Modification)
①	[24] (2022)	Coupling: dispersive coupling → dissipative coupling	NEP~81 Pa/Hz^1/2^; narrow bandwidth (limited by mechanical resonance)	NEP = 0.81 Pa/Hz^1/2^ (Exp.); Bandwidth = 30–800 kHz (Exp.)
②	[125] (2024)	Packaging: planar packaging → perforated hollow array	STL = 160 dB (Sim.); Low-frequency signal shielded	STL = 130 dB (Sim.); Sensitivity = −176.3 dB re 1 V/μPa @ 300 Hz (Exp.); NEP = 0.87 mPa/Hz^1/2^ @ 300 Hz (Exp.)
③	[156] (2024)	Readout: free-running → active frequency-locking	Photothermal signal ~0.1 a.u. (Exp.); peaks buried in noise	Photothermal signal ~1.0 a.u. (Exp.)

STL = sound transmission loss; a.u. = arbitrary units. Sim. denotes simulation results; Exp. denotes experimental measurements.

As illustrated in Table 7, switching from dispersive to dissipative coupling can substantially broaden operating bandwidth at the cost of absolute detection sensitivity. Optimized hollow-array packaging mitigates low-frequency acoustic signal shielding and improves acoustic transmission efficiency. Moreover, adopting active frequency-locking readout significantly raises signal amplitude and lifts signals out of the noise floor. These cases quantitatively demonstrate how coupling configuration, packaging architecture and readout schemes shape the final sensing performance.

**Figure 18:**
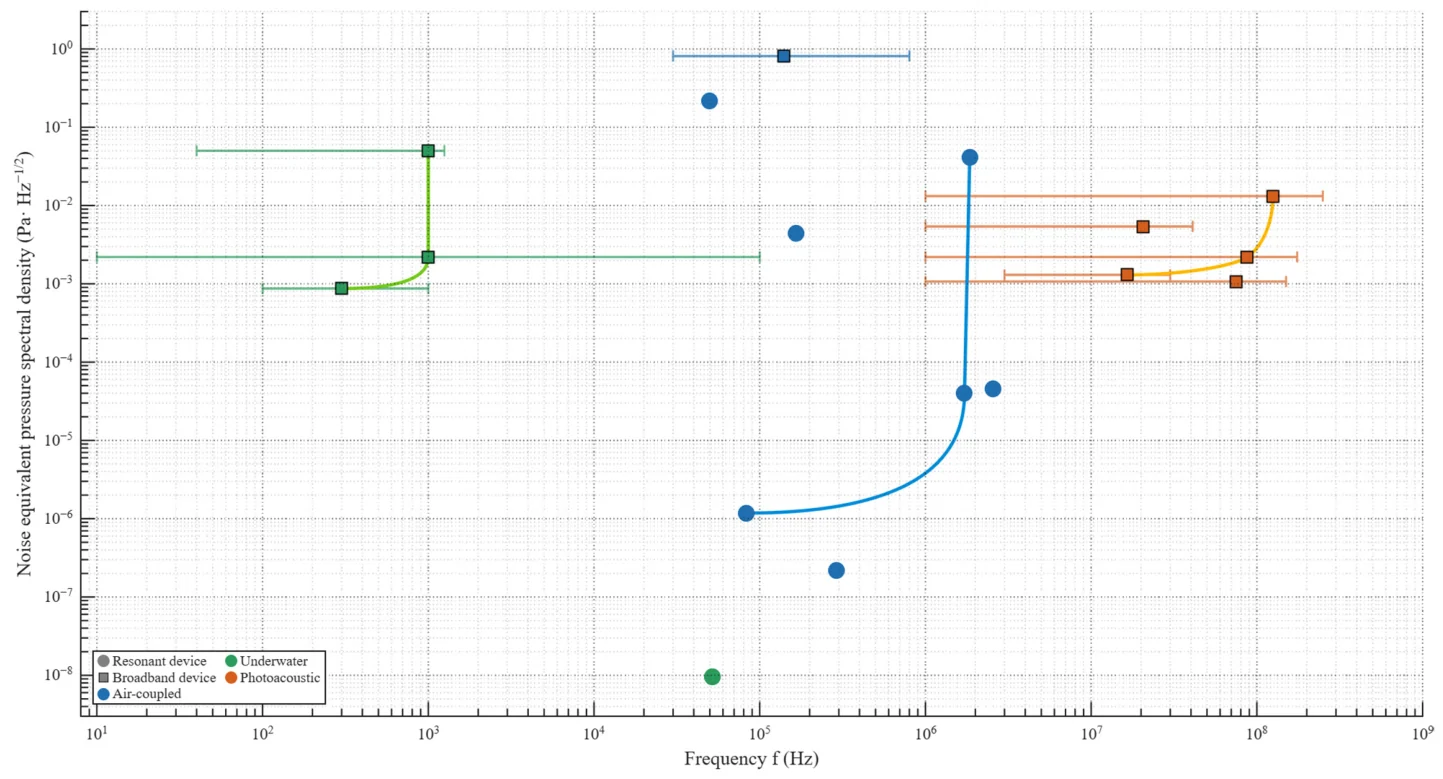
Noise-equivalent pressure (NEP) versus operating frequency for reported WGM optical microcavity acoustic sensors. Data points are extracted from cited references [16,17,18,24,25,26,52,53,58,81,125,126,136,137,148] reporting both NEP and working frequency. Circles denote resonant devices and squares broadband devices. Colors distinguish application scenarios: green for underwater, blue for air-coupled, orange for photoacoustic imaging. Horizontal bars represent the operational frequency bandwidth of sensors reported in original works; dashed rectangles mark references with interval NEP values. Solid curves are visual trend guides and do not represent the frequency response of any single sensor.

While Table 7 focuses on targeted single-variable comparisons for selected experiments, it is instructive to overview the overall performance distribution across the broader body of reported WGM microcavity acoustic sensor research. For this purpose, compiled noise-equivalent pressure-versus-frequency data from Table 3, Table 4 and Table 5 are plotted in Figure 18.

To summarize the device performance of WGM optical microcavity acoustic sensors discussed throughout this section, and to validate the frequency-dependent NEP relationship derived in Section 3.3, we collect all literature data points with explicitly reported noise-equivalent pressure and corresponding operating frequency, covering underwater, air-coupled and photoacoustic applications, as illustrated in Figure 18.

As observed in Figure 18, the compiled data span a broad frequency range from ~10^1^ Hz for underwater acoustic detection up to ~10^8^ Hz for photoacoustic imaging, with NEP ranging from 10^−8^ to 10^0^ Pa/Hz^1/2^. Resonant devices (circular markers) achieve lower NEP near their mechanical resonant frequency, yet this sensitivity gain comes with a narrow usable bandwidth. In contrast, broadband devices (square markers) maintain a relatively flat NEP over a wide frequency band, at the expense of higher noise floor. The horizontal bars extending from each data point indicate the operational frequency bandwidth of each sensor reported in the original literature. In terms of application scenarios, underwater sensors mainly work at low frequencies of 10^1^–10^5^ Hz; air-coupled sensors concentrate in the mid-frequency band of 10^5^–10^6^ Hz; photoacoustic sensors operate at high ultrasonic frequencies (10^6^–10^8^ Hz), mostly adopting broadband structures. This distribution trend agrees with the theoretical NEP(*f*) model in Equation (12). Resonant enhancement reduces NEP around mechanical resonance $f_{m}$, with bandwidth approximated as $\Delta f\approx f_{m}/Q_{m}$. When operating far below mechanical resonance, broadband sensors exhibit flat frequency response but degraded sensitivity. This figure demonstrates the inherent sensitivity–bandwidth trade-off of WGM microcavity acoustic sensors, which is the core limitation restricting further performance improvement.

## 5. Challenges and Outlook

### 5.1. Existing Challenges

Although remarkable progress has been achieved in WGM optical microcavity acoustic sensing, several technical challenges remain in calibration, environmental stability, packaging and scalable readout. These challenges arise from the structures and materials discussed in Section 2, the response and noise mechanisms in Section 3, and the medium adaptation and function extension reviewed in Section 4.

(1) Inconsistent calibration and performance reporting

Comparisons of reported NEP values require consistent definitions and documentation of acoustic frequency, noise bandwidth and normalization, operating medium, and readout conditions [48,127]. Differences in device architecture and pressure calibration procedures must also be considered. Existing free-field hydrophone calibration procedures provide a reference for underwater measurements [158], but their applicability to a given optical sensor configuration must be assessed. Consistent reporting of calibration uncertainty, frequency-dependent response and noise normalization is therefore needed to interpret the comparisons in Table 3, Table 4, Table 5, Table 6 and Table 7.

(2) Thermal drift, technical noise and cross-sensitivity

WGM resonances respond to temperature fluctuations, mechanical perturbations and refractive-index changes [29]. Thermo-optic and thermal expansion effects can shift the operating point and alter the acoustic readout gain. Laser frequency and intensity fluctuations can also introduce technical noise [128]. Self-referenced optical thermometry provides temperature information for stabilization [159], but its integration with acoustic readout requires evaluation of residual interference. In the multifunctional sensing configurations reviewed in Section 4.3, responses to different physical quantities must be distinguished before quantitative multiparameter decoupling can be claimed.

(3) Acoustic coupling and packaging trade-offs

Air-coupled detection requires acoustic coupling to be considered together with resonator geometry and mechanical response, as illustrated by shell resonator studies [25]. Underwater operation introduces additional packaging constraints. Hollowed-out encapsulation and polymer-clad microbubble designs demonstrate approaches to maintaining acoustic transmission and optical operation [125,136]. Nevertheless, the effects of packaging materials and geometry on acoustic response and optical loss must be assessed for the intended frequency range. As discussed in Section 4.1, packaging therefore requires joint consideration of acoustic transmission, the preservation of the optical Q factor and environmental protection.

(4) Long-term operational stability

Packaged WGM microprobes [53], longitudinal photoacoustic imaging over 28 days [147], and field-validated underwater sensors [121] demonstrate progress towards practical operation. These demonstrations do not replace evaluation of calibration drift and coupling stability under the conditions required for each measurement task. For prolonged operation, adhesive aging, surface contamination and structural relaxation should be evaluated as potential failure mechanisms where applicable. The practical challenge is to establish how changes in the optical operating point affect calibrated acoustic performance over time.

(5) Fabrication repeatability, array uniformity and scalable readout

Low-loss Si_3_N_4_ manufacturing provides a route towards scalable fabrication [86], while fiber-facet integration can reduce assembly demands [58]. However, variations in optical resonance and coupling conditions can complicate array calibration. A 15-element microring array has demonstrated parallel photoacoustic interrogation using one light source and one photoreceiver [81]. Further scaling in this architecture is constrained by interrogation bandwidth, resonance separation, drift and demodulation requirements [81]. The challenge is therefore to extend the array capabilities reviewed in Section 4.2 while maintaining channel consistency and manageable readout complexity.

### 5.2. Future Outlook

Building on these challenges, future research should connect the response and noise analysis in Section 3 with the requirements of air-coupled sensing, liquid-phase sensing, photoacoustic imaging and multifunctional sensing in Section 4. The following directions emphasize measurable improvements and their validation.

(1) Establish traceable calibration and consistent evaluation protocols

Calibration should use a documented reference pressure field and report measurement uncertainty, drawing on established hydrophone procedures where applicable [160]. Complete test documentation should specify the medium, acoustic frequency, calibration method, equivalent noise bandwidth, optical power, readout operating point and environmental conditions. Noise spectra and response functions should be reported together so that frequency-dependent NEP can be evaluated [48]. Integrated pressure thresholds should be distinguished from NEP, and values reported at one frequency should not be interpreted as performance across the full band. These practices would strengthen the comparisons in Table 3, Table 4, Table 5, Table 6 and Table 7 and the NEP–frequency plot.

(2) Improve environmental robustness through stabilization and packaging

Self-referenced temperature monitoring could be combined with resonance stabilization to track operating point drift [159]. Laser frequency and intensity noise should be characterized at the readout operating point to identify whether technical noise limits acoustic detection [128]. Packaging optimization should be guided by the transmission and environmental stability results discussed in Section 4.1 [125,136]. Building on longitudinal imaging and field validation studies [121,147], future prototypes should be evaluated for changes in calibrated response after immersion, mechanical disturbance and repeated temperature or pressure changes, as appropriate to the intended operating conditions. This would connect short-term NEP measurements with sustained measurement reliability.

(3) Develop reproducible resonator arrays and scalable interrogation

Extending the arrays reviewed in Section 4.2 to two-dimensional layouts could increase spatial sampling for photoacoustic imaging; three-dimensional arrangements of packaged probes could support acoustic field sampling at multiple depths. These are prospective architectures whose coupling access, element spacing and mutual acoustic perturbations require validation. Coherent signal combination may improve signal-to-noise ratio when the relevant noise is uncorrelated, but does not by itself extend each channel’s linear dynamic range. Complementary channel gains or operating points could be investigated for range extension, with overlap calibration and saturation tests. For fabrication, lithographically defined resonators and waveguides [26,86] and directly written fiber-facet coupling bridges [58] offer routes to reducing repeated manual alignment. Automated positioning and fixation should be evaluated using coupling loss, assembly yield and repeatability. Digital optical frequency comb interrogation already provides a parallel-readout example [81]; increasing channel count requires control of resonance spacing, drift, crosstalk and data processing load. Array gains should be assessed under stated optical-power, bandwidth and acquisition time conditions.

(4) Intelligent signal processing for target identification and multiparameter decoupling

Building on Section 4.3 and Table 6, intelligent signal processing could extend WGM sensing from signal acquisition towards target identification. Liao et al. demonstrated machine learning-assisted classification of photoacoustic signals [80]. Hajihashemi et al. reviewed Mel-based feature extraction for acoustic event detection and classification, providing guidance on feature selection and parameter configuration [160]. For WGM signals, candidate Mel spectrograms could be obtained by applying a Mel filterbank to short-time Fourier power spectra; extraction of Mel frequency cepstral coefficients (MFCCs) additionally involves logarithmic compression and a discrete cosine transform. Their suitability should be compared with linear-frequency and other task-specific representations, particularly for ultrasound. Models should be tested on independent data spanning device and operating condition variations. Classification accuracy alone does not establish quantitative multiparameter decoupling: simultaneous perturbations require independently calibrated measurands and complementary sensing information. Joint optimization of device readout and signal interpretation could therefore connect the multifunctional capabilities reviewed in Section 4.3 with validated measurement tasks.

## 6. Conclusions

Whispering-gallery-mode (WGM) optical microcavity acoustic sensors possess prominent merits including high sensitivity, a broad detection bandwidth, a compact footprint and immunity to electromagnetic interference, rendering them a competitive alternative to conventional piezoelectric and MEMS acoustic transducers. In this review, we systematically introduced two fundamental acousto-optic sensing paradigms for WGM microcavities: dispersive coupling and dissipative coupling. Within the framework of perturbation theory, we established a unified theoretical description to distinguish the optical quality factor $Q_{0}$ and mechanical quality factor $Q_{m}$, and elucidated the intrinsic sensitivity–bandwidth trade-off imposed by the mechanical dynamics of microresonators, providing unified evaluation criteria for interpreting experimental sensing performances.

From the perspective of medium adaptation and function extension, this review summarizes the state-of-the-art research progress of WGM-based acoustic sensors for air-coupled and liquid-phase underwater detection. Resonance-enhanced sensors relying on dispersive coupling are capable of achieving ultra-low noise-equivalent pressure (NEP), yet their effective operating bandwidth is inherently limited by mechanical resonant modes. By contrast, sensors operated via dissipative coupling can deliver a broadband acoustic response, which is usually obtained at the cost of absolute detection sensitivity under practical coupling conditions. For liquid-phase detection scenarios, protective packaging introduces multi-objective conflicts among acoustic transmission efficiency, preservation of high optical $Q_{0}$ and long-term mechanical durability. We further surveyed representative advances in photoacoustic imaging and multiparameter multifunctional sensing. Quantitative comparisons of device performances under single-variable optimization are summarized in Table 7, while the compiled NEP–frequency scatter plot demonstrates good consistency between experimental observations and the theoretical noise–bandwidth model derived in this work.

Nevertheless, multiple practical bottlenecks still hinder the translation of laboratory prototypes toward real-world engineering deployment. Traceable and unified acoustic-pressure calibration standards are still absent in this community. Thermal drift, laser technical noise and multi-physical-field crosstalk severely degrade measurement reliability. Conflicting constraints between acoustic impedance matching and packaging design remain nontrivial challenges. In addition, long-term operational stability, the fabrication repeatability of microcavity devices, array-element uniformity and scalable multi-channel readout architectures have not been fully resolved.

Future investigations call for joint optimization covering hardware configuration, manufacturing processes and post-signal processing algorithms. Standardized calibration and performance reporting protocols should be formulated to facilitate fair comparison among different sensor prototypes. Combined hardware–algorithm solutions need to be further explored to suppress environmental interference. Automated coupling techniques and parallel interrogation architectures are urgently required to promote the implementation of two-dimensional and three-dimensional microcavity sensor arrays. Moreover, deep learning approaches based on Mel spectral features offer promising prospects for multiparameter decoupling and acoustic event classification, serving as a powerful supplementary tool for hardware-oriented optimization. With continuous technical innovations, WGM optical microcavity acoustic sensors hold great application promise in biomedical photoacoustic imaging, underwater acoustic monitoring and industrial non-destructive testing.

## Figures and Tables

**Table 1:** Comparison of research scopes of three WGM microcavity acoustic sensing reviews.

Reference	Research Objects	Summary of Main Contents
[48] (2024)	F-P cavities, π-BG, WGM microcavities for ultrasound sensing	① Dispersive/dissipative coupling mechanisms ② Microcavity principles and structures ③ Summary of key sensing parameters ④ Applications and multi-device comparison
[49] (2025)	Light–sound interaction in 3D WGM microcavities	① WGM, coupling and fabrication ② Theoretical modeling of microbubble resonators ③ Mode-based sensing mechanisms ④ Overview of diverse sensing applications
This work (2026)	WGM microcavity acoustic sensing	① Elaborate acoustic sensing mechanisms and readout mechanisms ② Analyze noise- and bandwidth-related performance limits based on the NEP metric ③ Review sensing advances for air-coupled and liquid-phase propagation media ④ Summarize photoacoustic imaging and multifunctional sensing applications

**Table 2:** Advantages and limitations of typical materials for WGM microcavity acoustic sensing.

Material Category	Typical Materials	Advantages	Limitations
Amorphous inorganic glass	SiO_2_	Low optical loss, potentially enabling ultra-high Q resonators [54]	Brittle, requiring careful packaging and handling
	Chalcogenide glass (As_2_S_3_)	High refractive index and broad infrared transparency	Thermo-optic effects may induce resonance shifts with temperature [84]
Inorganic dielectric	Si_3_N_4_	Supports low-loss, scalable photonic integration [86]	Achievable Q factor is sensitive to film morphology and side-wall roughness
Semiconductor	Si GaAs InP	Mature microfabrication facilitates on-chip integration [88,91,94]	Exhibits wavelength-dependent optical absorption and thermo-optic responses
Dielectric crystal	CaF_2_ MgF_2_ LiNbO_3_	Can achieve ultra-high Q; CaF_2_ is promising for acoustic detection [95,96]	Fabrication and optical coupling are technically demanding
Polymer	PMMA PS SU-8 PDMS	Flexible processing; PDMS enables deformable cavities [112]	Optical loss and viscoelasticity vary with material and structure [106,109]

**Table 3:** Representative performance of air-coupled WGM microcavity acoustic sensors.

Reference	Device Structure	Material	Frequency Range	Q Factor	NEP
[129] (2019)	Suspended spoked silica microdisk	SiO_2_	1 kHz–1 MHz	3.6 × 10^6^	8–300 μPa/Hz^1/2^ @ 1 kHz–1 MHz
[17] (2023)	Microdisk with etched trench structures	SiO_2_	~20 kHz–1 MHz+	~10^6^	1.18 μPa/Hz^1/2^ @ 82.6 kHz
[25] (2024)	On-chip microspherical shell resonator	SiO_2_	0.6–3.5 MHz	~2 × 10^6^	40 μPa/Hz^1/2^ @ 1.72 MHz
[26] (2026)	Suspended SiO_2_ membrane embedding Si_3_N_4_ microring resonator	SiO_2_, Si_3_N_4_	20 kHz–1 MHz	1.35 × 10^6^	218 nPa/Hz^1/2^ @ 289 kHz

**Table 4:** Representative performance of liquid-phase WGM microcavity acoustic sensors.

Reference	Device Structure	Material	Acoustic Medium	Frequency Range	Q Factor	Sensitivity	NEP	Packaging Scheme
[136] (2022)	Packaged microsphere cavity	SiO_2_	Water	10 Hz–100 kHz	10^8^ (before packaging); ~10^6^ (after packaging)	−159.3 dB re 1 V/μPa @ 1 kHz	2.2 mPa/Hz^1/2^ @ 1 kHz	MY-133-MC optical coating
[137] (2023)	Ring optical waveguide resonator	SiO_2_	Water	40–1250 Hz	1.78 × 10^7^	−163.1 dB re 1 V/μPa @ 1 kHz	50 mPa/Hz^1/2^ @ 1 kHz	UV-cured adhesive fixation
[125] (2024)	Microdisk resonator	CaF_2_	Water	100 Hz–1 kHz	1.2 × 10^8^	−176.3 dB re 1 V/μPa @ 300 Hz	0.87 mPa/Hz^1/2^ @ 300 Hz	Perforated hollow-out array packaging
[26] (2026)	Suspended SiO_2_ membrane-embedded Si_3_N_4_ microring resonator	SiO_2_, Si_3_N_4_	Water	20 kHz–1 MHz	1.33 × 10^6^ (in water)	—	9.6 nPa/Hz^1/2^ @ 52 kHz	SiO_2_ layer encapsulation
[121] (2026)	Microtoroid resonator	SiO_2_	Indoor water tank/lake water/seawater	100 Hz–100 kHz	Decreased from 1 × 10^7^ to 1 × 10^6^ after polymer coating	−156.95 dB re 1 V/μPa @ 60 kHz (indoor water tank)	—	MY-132A polymer coating

**Table 5:** Representative performance of WGM microcavity-based photoacoustic imaging devices.

Reference	Device Structure	Material	Q Factor	Bandwidth	NEP	Lateral Resolution	Axial Resolution	Array Count	Packaging Scheme
[147] (2019)	Microring	PS	1.4 × 10^5^ (pristine); 3.6–4.2 × 10^4^ (28-day in vivo)	—	—	~4 μm	—	1	PDMS encapsulation
[148] (2021)	Microring 1D array	Si	~10^4^	3–30 MHz	1.3 mPa/Hz^1/2^	—	—	10	—
[81] (2023)	On-chip Microring 1D array	chalcogenide	5 × 10^5^–7 × 10^5^	175 MHz (−6 dB)	2.2 mPa/Hz^1/2^ (single-channel); 13.9 mPa/Hz^1/2^ (DOFC readout)	50.4 μm	43.6 μm	15	PDMS encapsulation
[58] (2024)	Microring	IP-L photoresist	6 × 10^4^	103 MHz (−6 dB); >250 MHz response	13.2 mPa/Hz^1/2^	5 μm	6.6 μm	1	MY-133MC low-RI adhesive encapsulation

## Data Availability

No new data were created or analyzed in this study. Data sharing is not applicable to this article.
